# Behavioral variability and neural cross-frequency coupling distinguish working memory maintenance from manipulation

**DOI:** 10.1162/IMAG.a.1155

**Published:** 2026-02-27

**Authors:** Yingzhe Li, Romy Lorenz, Guang Ouyang

**Affiliations:** Complex Neural Signals Decoding Lab, Faculty of Education, The University of Hong Kong, Hong Kong Island, Hong Kong; Cognitive Neuroscience & Neurotechnology Group, Max Planck Institute for Biological Cybernetics, Tübingen, Germany; Department of Neurophysics, Max Planck Institute for Human Cognitive and Brain Sciences, Leipzig, Germany

**Keywords:** working memory, cross-frequency coupling, cross-individual variability, maintenance, manipulation

## Abstract

Working memory (WM) is typically understood to comprise two core components: maintenance (short-term retention) and manipulation (active processing of information). However, many existing WM tasks are composite in design, involving both components and even other cognitive processes or constructs. Explicit characterization of the two components in terms of cross-individual variability in both behavioral performance and neural correlates remains limited. To address this gap, we developed two structurally matched task paradigms to compare the behavioral variability in WM maintenance and manipulation while minimizing extraneous influences such as strategy use and domain-specific skills. We also used phase-amplitude cross-frequency coupling (CFC) as a neural measure to investigate the neural dynamics of each WM component. Our psychometric analyses converged in supporting the differentiation between the two components. In addition, neural evidence showed that they engaged different brain networks. Specifically, WM manipulation recruited more extensive networks, reflected in stronger and structurally distinct phase-amplitude CFC across brain regions. Moreover, individual differences in manipulation task performance were reliably predicted by the corresponding CFC patterns, whereas maintenance task performance was not, further highlighting the two components’ differential association with neural dynamics.

## 1 Introduction

Working memory (WM) is commonly defined as the ability to retain and manipulate information in a highly accessible state over a short period (Crone et al., 2006; Eriksson et al., 2015). It plays a foundational role in human cognition, supporting problem-solving, reasoning, decision-making, and learning (Cowan, 2014; Hinson et al., 2003; Süß et al., 2002; Wiley & Jarosz, 2012). Despite its importance, WM remains a multifaceted construct, sparking ongoing debates about its conceptualization, structure, and measurement (Baddeley, 2012; Brady et al., 2016; Cowan, 2017; Oberauer, 2019; Vandierendonck, 2021; Wilhelm et al., 2013). Key unresolved issues include its composition (Baddeley, 2012; Cowan, 2010; Oberauer, 2009) and how it can be functionally distinguished from related constructs like attention (Chun et al., 2011; Gazzaley & Nobre, 2012; Kiyonaga & Egner, 2013).

Despite persistent open questions about WM at the mechanistic level, one key theoretical consensus that has been reached in recent years is that WM consists of two primary functional components: information maintenance and manipulation (or operation)—one refers to retaining information over a short period for current use while the other one refers to the active processing and operation of this live information (Aben et al., 2012; Baddeley, 1996; D’Esposito et al., 1999; Eriksson et al., 2015; Glahn et al., 2002; Masse et al., 2019; Vergauwe et al., 2014). This framework is conceptualized based on an information-computational perspective, but its detailed psychometric characterization at both behavioral and neural levels remains limited.

In this work, we aimed to design well-controlled WM measurement tasks to characterize cross-individual variability in both WM components at behavioral and neural levels. Regarding the task design, we identified some limitations in current approaches to WM measurement. These limitations are rooted in the diversity and complexity of existing WM tasks, many of which introduce potential confounding factors that may compromise measurement validity. A common limitation is domain-specific skill dependence, where performance is affected by external abilities such as arithmetic in the operation span task (Turner & Engle, 1989). Another limitation is strategy reliance, where participants use shortcuts like associative memory (Jones & Macken, 2015; Stuart & Hulme, 2000) or subvocal rehearsal. While the latter (subvocal rehearsal) occurs naturally in verbal WM (Baddeley, 2000), it plays a limited role in conceptual processing and does not reflect the demands of real-world WM use (Cowan, 2001). Moreover, its repetitive and self-paced nature engages neural mechanisms distinct from general information storage (Awh et al., 1996; Hwang et al., 2005). A third limitation is interference from other cognitive processes: for instance, the n-back task (Kirchner, 1958) strongly depends on sustained attention, which may contaminate its measurement of WM if attention is treated as a separate construct.

Besides the issue of diversity and complexity in existing WM measurement, we have not found a psychometric measurement framework that explicitly distinguishes between maintenance and manipulation components while holding all other task features constant. Although prior psychometric studies using structural equation modeling (SEM) have examined the relationship between WM tasks (which typically involve both maintenance and manipulation) and short-term memory tasks (which primarily reflect maintenance), and have reported evidence of differentiation (e.g., Engle et al., 1999; Swanson & Kim, 2007), these studies often used tasks with considerable content variation. Such variation may itself contribute to the observed differences. A more effective approach would involve a unified paradigm in which two structurally identical tasks differ only in whether they require manipulation in addition to maintenance, which has not been explored in behavioral data-based psychometric studies.

In the neuroscience field, a large body of neuroimaging research has consistently identified distinct neural signatures between WM maintenance and manipulation based on unified paradigms (D’Esposito et al., 1999; c.f. Berger et al., 2019; Wager & Smith, 2003). However, while unified paradigms have been used in neuroscientific studies, they have primarily targeted neural correlates rather than individual differences in WM maintenance and manipulation. For example, in neuroscientific studies adopting a unified paradigm based on the n-back task (e.g., Jablonska et al., 2020; Seo et al., 2014; Veltman et al., 2003), the 1-back condition was used to assess maintenance, and the 2-back condition was used to assess both maintenance and manipulation of WM. However, the 1-back task may not effectively measure maintenance ability in behavioral studies due to ceiling effects, limiting its ability to capture meaningful individual differences. Although neuroscientific research has shown separable substrates for maintenance and manipulation (e.g., D’Esposito et al., 1999; Glahn et al., 2002; Mohr et al., 2006; Veltman et al., 2003), few studies have assessed their differences through factor analysis or SEM at the level of cross-individual behavioral or neural variability.

A further challenge we considered lies in the frequent confounding of speed and accuracy. In difficult tasks, response speed may no longer reflect processing efficiency on its own, as participants tend to slow down to avoid errors, making speed and accuracy interdependent rather than independent indicators (Draheim et al., 2016; Heitz, 2014). At the same time, accuracy-based measures are susceptible to influences not directly related to WM function, such as attention lapses, motivational fluctuations, or anxiety. These challenges are further complicated by task difficulty: ceiling effects in easier conditions and greater variability in harder ones can distort performance patterns and reduce the interpretability of both measures (Meule, 2017).

Considering the identified issues outlined above, we developed a new task paradigm designed to compare maintenance and manipulation, while minimizing the influence of domain-specific biases, strategy reliance, other confounding cognitive abilities, and the confounding between speed and accuracy. The task paradigm includes two structurally identical sub-tasks: a maintenance task and a manipulation task, differing only in that the latter requires reordering the information in addition to maintaining it. In the maintenance task, participants viewed three images presented sequentially and recalled their chronological order by button-clicking. In the manipulation task, the setup was identical, but participants were required to reorder the images according to a designated sequence, thus entailing an additional layer of information manipulation. The images used were abstract and devoid of semantic content, reducing the potential influence of domain-specific knowledge or skills. This design aimed to minimize such domain-specific cognitive confounds and to limit excessive reliance on specific strategies, such as subvocal rehearsal (Phillips & Baddeley, 1971). The task difficulty was calibrated so that accuracy remained high, thereby allowing individual differences to be reflected mainly in response speed.

Based on this design, we expect that the behavioral performance data obtained from the two tasks will reflect observable differential cross-individual variabilities of WM maintenance and manipulation. Moreover, we also expect that the behavioral indicators from multiple trials within each task are relatively homogeneous, which will be reflected by factor analysis showing high loadings of separate indicators to the same factor. A high homogeneity indicates less blending of unrelated cognitive processes (e.g., complex domain-specific skills) or speed-accuracy mixtures. To evaluate the degree to which task design and content determine the construct measured, we compared our WM measurements with a well-established WM task—the operation span task, which correlates strongly with higher-order cognitive abilities such as reasoning and fluid intelligence (Kane et al., 2004; Unsworth & Engle, 2005).

To further validate our findings, we examined whether the neural correlates of WM processes also reflect structural differences between maintenance and manipulation. We expected differences in neural indicators at two levels. First, the neural dynamics should reveal the differentiation between the two WM components, as indicated by divergent patterns of brain responses and connectivity. Second, cross-individual variability in these neural measures, paralleling behavioral data, would also reflect differences in their underlying neural substrates.

We employed electroencephalography (EEG) to capture the temporal neural dynamics during the task and analyzed phase-amplitude cross-frequency coupling (CFC) to investigate how these processes are coordinated in the brain. Phase-amplitude CFC involves the modulation of high-frequency amplitude by the phase of lower frequency oscillations (Canolty & Knight, 2010). This mechanism plays a crucial role in neural computation and communication, supporting complex cognitive functions such as WM, cognitive control, and learning (Canolty & Knight, 2010; Riddle et al., 2021; Roux & Uhlhaas, 2014). Given that WM depends on the efficient coordination of distributed neural processes, CFC provides a mechanistic perspective on how different aspects of WM function are integrated across brain regions. Prior research has linked CFC to both WM maintenance (Axmacher et al., 2010; Daume et al., 2024; Roux & Uhlhaas, 2014) and information processing such as WM manipulation (Canolty & Knight, 2010; Miller et al., 2018). For these reasons, we examined phase-amplitude CFC as a neural marker of the mechanisms underlying WM maintenance and manipulation.

Since CFC is closely tied to information processing, and given that neural computation is expected to differ between manipulation and maintenance, we hypothesized that the phase-amplitude CFC profile would distinctly reflect these two WM components. First, the CFC in manipulation would be significantly stronger because it involves more complex information processing that requires more demanding neural computation and coordination. Second, we anticipate that CFC will reflect the distinction between the two WM components in cross-individual variability. Third, because CFC can be calculated across electrodes, it may reveal differences in processing networks between maintenance and manipulation, indicating qualitatively distinct underlying mechanisms.

## 2 Methods

### 2.1 Experiment design

#### 2.1.1 Participants

A total of 68 participants aged 18 to 30 years (41 females, 27 males; mean age 24.25 years) from the University of Hong Kong completed the experimental tasks. All participants reported normal or corrected-to-normal vision and no history of neurological disorders. The study was approved by the Human Research Ethics Committee (HREC) of the University of Hong Kong. Four participants were excluded from all analyses: three excluded due to low task accuracy (below 80% in both WM maintenance and manipulation tasks; see Section 2.2.4) and one excluded by the outlier criterion of being larger than 4 standard deviations (SD) from the group average. An additional participant was excluded from the neural analyses because ocular artifacts could not be adequately removed from the EEG recordings.

#### 2.1.2 Apparatus and EEG recording

The experiments were conducted in a quiet, dimly lit room, where participants performed a series of tasks while EEG data were recorded. EEG signals were recorded using a 32-channel BrainAmp DC amplifier (Brain Products, Germany), referenced to Fpz, with a sampling rate of 1000 Hz. The data were stored using BrainVision Pycorder. The electrodes were mounted on an elastic cap (Easycap, Brain Products, Germany) according to the international 10–20 system (Jasper, 1958). Stimuli were presented on an LCD monitor with a 1920 × 1080-pixel resolution, viewed from a distance of approximately 60 cm.

#### 2.1.3 Experimental task

**Fig. 1.**
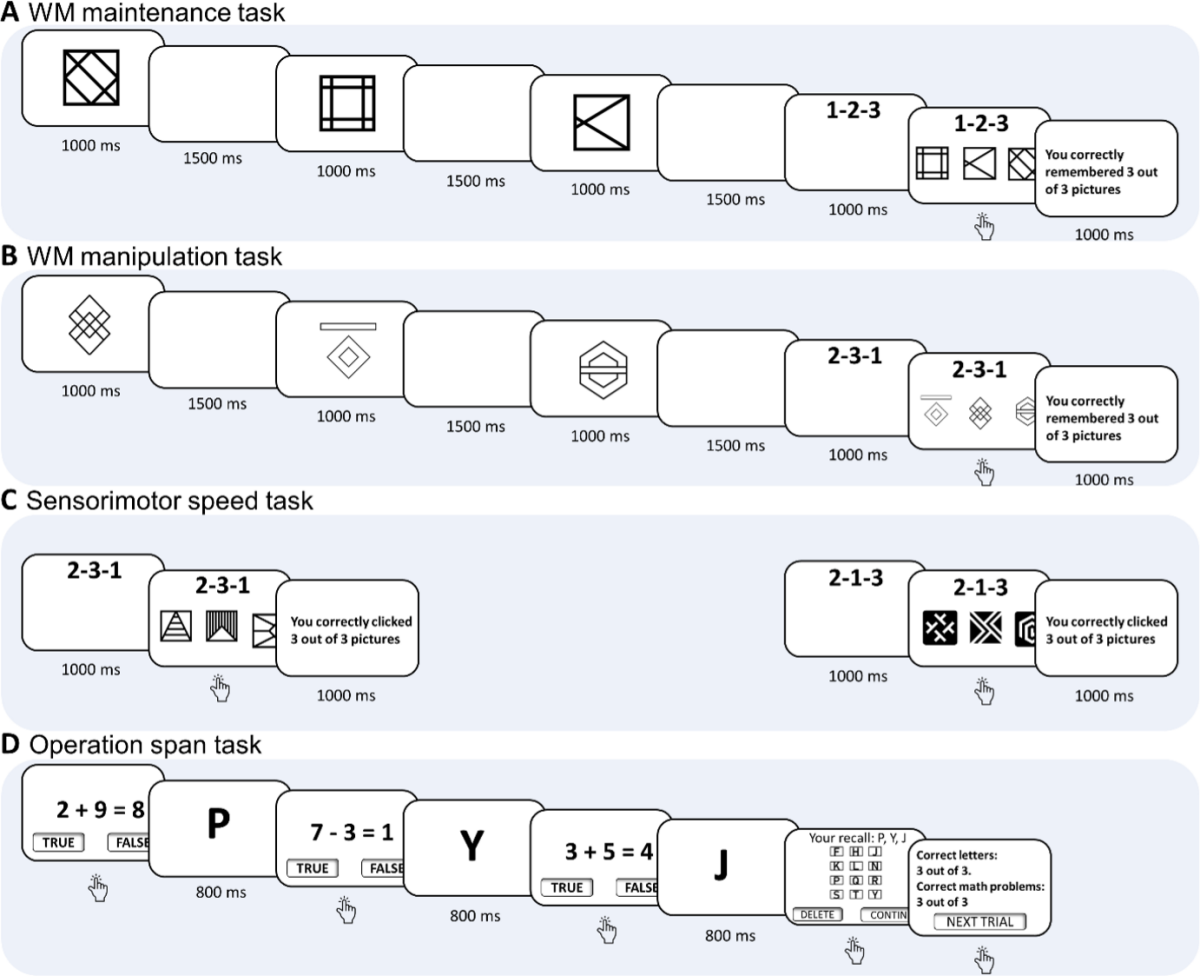
Schematic diagrams outlining trial(s) for each task. (A) WM maintenance task, (B) WM manipulation task, (C) sensorimotor speed task, and (D) operation span task.

Four experimental tasks were administered in the current study (see Fig. 1). Three of these tasks formed a composite of our newly designed paradigm: the WM maintenance task, WM manipulation task, and sensorimotor speed task. The fourth task, namely the operation span task, was used for comparison with our newly designed paradigm. The order of administering the composite WM tasks and the operation span task was counterbalanced across participants. All tasks collected participants’ behavioral performance by requiring them to click the mouse button to make their selections.

The WM maintenance task was designed to focus on assessing performance in maintaining information, whereas the WM manipulation task added a layer of information manipulation on top of the WM maintenance task. To minimize potential variability in neural indicators caused by fluctuations in participants’ mental states and ensure valid EEG comparisons, the WM maintenance and WM manipulation tasks were administered together in a counterbalanced order.

Before entering the main experiment, participants completed two practice trials for each task. To proceed to the main experiment, they were required to achieve full accuracy in these practice trials, ensuring that they fully understood the tasks.

##### 2.1.3.1 WM maintenance task

In the WM maintenance task, participants were sequentially presented with three abstract images, each displayed for 1 second, followed by a 1.5-second inter-stimulus interval. Subsequently, a sequence prompt displaying ‘1-2-3’ appeared for 1 second, instructing participants to click the first, then the second and third images they just saw in the subsequent frame where the three images were presented in a randomized order. The prompt ‘1-2-3’ was redundant in this task but was included to ensure consistency in stimulus input with the manipulation task, where the sequence varies across trials. After participants’ selection, feedback was given to indicate how many images were correctly clicked in order, and stayed on the screen for 1 second, followed by a 1-second intertrial interval before the next trial began.

##### 2.1.3.2 WM manipulation task

The basic structure of the WM manipulation task mirrors that of the WM maintenance task, with the only difference lying in the sequence prompt. In the WM maintenance task, the sequence prompt is always ‘1-2-3’, requiring participants to sequentially click the images in the original order of presentation. However, in the WM manipulation task, participants were required to follow a variable sequence prompt, chosen from one of four designated sequences (‘2-1-3’, ‘2-3-1’, ‘3-1-2’, and ‘1-3-2’). Each sequence necessitated reordering the original image sequence before clicking, adding an active processing step beyond the simple maintenance of information. While ‘3-2-1’ is also a possible sequence following the design standard, it was not used due to its relatively low difficulty.

The visual stimuli used in both WM tasks consisted of abstract images devoid of semantic content, selected from the open-access Flaticon library (https://www.flaticon.com/packs) using the keyword “abstract”. The icons were black-and-white vector graphics without representational or color elements, minimizing potential semantic associations. A total of 120 unique images were used, grouped into 40 distinct three-image sets. Within each set, the three images were visually comparable in layout, complexity, and component structure to maintain consistent perceptual demands across trials. Each image was displayed at a size of 200 × 200 pixels and positioned horizontally at −300, 0, and +300 pixels relative to the screen center, with the order randomized on each trial. At a typical viewing distance of approximately 60 cm, each image measured about 5° of visual angle, with centers located at roughly −7°, 0°, and +7° horizontally.

Each participant completed a total of 40 trials in both the WM maintenance and manipulation tasks. To account for potential biases caused by specific images, the image sets were systematically alternated and counterbalanced between the two tasks across participants.

##### 2.1.3.3 Sensorimotor speed task

To evaluate the contribution of sensorimotor speed to the WM measurements, a sensorimotor speed task was included. In this task, participants clicked on images in a designated sequence, providing a baseline measurement of sensorimotor speed. The basic structure of this task mirrors that of the WM maintenance and WM manipulation tasks, except that no stimuli were presented for memorization. Participants were presented with a sequence prompt for 1 second, followed by the simultaneous display of three images in a row, which is consistent with the format used in the WM maintenance and manipulation tasks. They were instructed to click on the images in the order specified by the sequence prompt, with the leftmost image designated as ‘1’, the middle image as ‘2’, and the rightmost image as ‘3’. After selection, feedback indicating the accuracy was presented for 1 second, followed by a 1-second intertrial interval before the start of the next trial. This task included 15 trials, with three trials corresponding to each of the five designated sequences used in the WM maintenance and manipulation tasks.

##### 2.1.3.4 Operation span task

The operation span task was based on the standard protocol outlined by Unsworth et al. (2005). In this task, participants were required to solve a mathematical operation task followed by the memorization of a letter. Each block of the task contained a series of equation-letter pairs presented in varying set sizes of 3, 4, 5, 6, or 7, where set size refers to the number of equation-letter pairs that participants needed to process and recall. The task consisted of three blocks, with a total of 15 trials. The order of set sizes was randomized across the entire experiment to minimize order effects.

During each trial, participants first saw an addition or subtraction equation involving integers ranging from 1 to 20, accompanied by a prompt requiring them to judge the equation as true or false. Unlike the original design, in which the equation included a combination of multiplication/division paired with subtraction/addition, we only used subtraction and addition to minimize the influence of math proficiency on task performance. Following the equation, a letter was presented for 800 milliseconds, which participants were instructed to memorize. This sequence was repeated according to the set size of that particular trial (3 to 7).

At the end of each trial, participants were presented with a 3*4 matrix of letters on the screen and were required to recall the letters in the order of their presentation by clicking on the corresponding letters in the matrix. The task was self-paced, and participants clicked the “next trial” button to proceed after receiving feedback on their accuracy in solving the math problems and recalling the letters.

### 2.2 Data analysis

#### 2.2.1 EEG preprocessing

The EEG recordings were pre-processed and analyzed using MATLAB and the EEGLAB Toolbox (Delorme & Makeig, 2004). The pre-processing steps followed the protocol published in Ouyang and Li (2025). Specifically, for each participant the steps included: (1) Down-sampling the EEG data to 250 Hz; (2) Applying an EEGLAB in-built high-pass FIR filter with a low cutoff of 1 Hz and a high cutoff of 100 Hz, followed by a notch filter to remove 50 Hz power-line noise; (3) Interpolating channels with missing or abnormally noisy data (e.g., disconnected channels); and (4) Selecting a segment of stationary EEG data that was visually inspected to contain no abnormal large-amplitude artifacts (e.g., abrupt transient fluctuation that may reach hundreds or thousands of microvolts) but only ocular artifacts and then applying independent component analysis to disentangle the EEG signal into independent sources. This step aimed to minimize the impact of high-amplitude transient artifacts on the independent component analysis (ICA) process and was followed by applying ICLabel to identify and remove ocular artifact components with a probability exceeding 0.8 (Pion-Tonachini et al., 2019). (5) After ocular artifact removal, segments of EEG data with amplitude exceeding ±100 µV (unlikely to be brain signals; most of them were due to temporal electrode disconnection or cap dragging) were inspected, and principal component analysis (PCA) was applied. The components with the highest variance were iteratively removed until the global field power of the reconstructed data fell within two median absolute deviations (MAD) from the clean data segments.

#### 2.2.2 Phase-amplitude cross-frequency coupling (CFC)

The phase-amplitude CFC between low and high-frequency bands in this study was characterized using two primary methods. First, to visualize the phase-amplitude CFC, the time-frequency representation (TFR) of each 1-second epoch was calculated using wavelet transformation based on Morse wavelet with the symmetry parameter (gamma) equal to 3 and the time-bandwidth product equal to 60. The magnitudes of the complex values obtained from the continuous wavelet transform (CWT) were used as the TFR, which was then averaged across epochs to reveal the modulation of high-frequency amplitude by the low-frequency phase. Before averaging, the TFRs from epochs with different phases were synchronized by shifting the TFRs according to their low-frequency phase values. Epochs with earlier phases were moved rightward, and those with later phases were moved leftward. This phase alignment enabled the examination of how the amplitude of high-frequency activity is modulated by the phase of low-frequency activity after averaging the synchronized TFRs (Tort et al., 2009).

After visualizing the phase-amplitude CFC, the next step was to quantify its strength using the modulation index (MI), following the procedures described in Tort et al. (2010). (1) The preprocessed EEG signals were filtered into two distinct frequency bands: one low-frequency band to extract the phase information, and one high-frequency band to extract the amplitude information. (2) The Hilbert transform was applied to these filtered signals to separately obtain the low-frequency phase and the high-frequency amplitude. (3) The high-frequency amplitudes were then discretized into 18 bins according to the corresponding low-frequency phases. (4) The MI was calculated by measuring the divergence of the amplitude distribution across these bins from a uniform distribution, using the formula provided below. The MI quantifies the extent to which the distribution of high-frequency amplitudes deviates from uniformity, where uniformity would indicate no phase-amplitude coupling. A greater divergence from a uniform distribution indicates a stronger modulation of high-frequency amplitude by the low-frequency phase.

$$\begin{array}{l} MI=\frac{D_{KL\;}(P,\;\;U)}{\log(N)}=\frac{\log(N)-\;H(P)}{log(N)}\\ \;\;\;\;\;\;\;\;\;\;=\frac{\log(N)-\sum_{j=1}^{N}P\left(j\right)log[P\left(j\right)]}{log(N)} \end{array}$$ (1)

$$P\left(j\right)=\frac{<\;A_{f_{A}}\text{​}{>}_{\varphi_{f_{p}}\left(j\right)}}{\sum_{k=1}^{N}<\;A_{f_{A}}\text{​}{>}_{\varphi_{f_{p}}\left(k\right)}}$$ (2)

where N is the number of phase bins, and $D_{KL\;}(P,\;U)$ denotes the Kullback-Leibler (KL) divergence between the observed amplitude distribution P and the uniform distribution U. The Shannon entropy *H(P)* denotes the uncertainty in the observed amplitude distribution. The frequency ranges $f_{p}$ and $f_{A}$ are used to extract the phase of the low-frequency band and amplitude information of the high-frequency band, respectively, with the phase time series $\varphi_{f_{p}}(t)$ and the amplitude time series $A_{f_{A}}(t)$ being derived from the Hilbert transform of the corresponding frequency bands. The mean amplitude value within each phase bin *j*, denoted as $<\;A_{f_{A}}\text{​}{>}_{\varphi_{f_{p}}\left(j\right)}$, is used to calculate the observed amplitude distribution P(j). Specifically, P(j) is calculated as $<\;\;A_{f_{A}}\text{​}{>}_{\varphi_{f_{p}}\left(j\right)}$ divided by the sum of mean amplitude values across all phase bins. The frequency range for obtaining the amplitude value was set to 40–80 Hz (Gamma band), while the frequency range for obtaining the phase value was set to 4–8 Hz (Theta band). These two frequencies were chosen as they were shown to be the two bands coupling each other in the time-frequency visualization plots (see above). We also considered choosing the bands based on individual data, but the time-frequency visualization of the CFC at the individual level was too noisy to allow proper selection of the frequency bands for each individual.

The time window for calculating the CFC began 500 ms after the prompt for image selection (to exclude sensory processing activity) and ended when participants clicked the second button (once the second click was made, the third click was considered predetermined and thus mainly reliant on sensorimotor processes). Data segments from each trial were concatenated. To construct the latent variables for the factor analysis, the MI values were first partitioned into three segments, each further binned into 5-second bins (a time window that generated more stable MI values); the final bin was discarded if shorter than 5 seconds. MI was computed for each bin and was averaged. The MI values from each of the three segments served as indicators for factor analysis later.

The MI algorithm and calculation described above can be conducted in two ways: within-electrode and cross-electrode. For within-electrode MI, a single value represents how the low-frequency phase modulates the high-frequency power at the same site. For cross-electrode MI, two values represent the modulation of low-frequency phase from one electrode to high-frequency amplitude at another. Here, we defined the directionality as the electrode providing low-frequency phase pointing to the electrode providing high-frequency amplitude for the CFC calculation. For each pair of electrodes, both directions can be calculated, resulting in two MI values. We used this directionality to characterize the difference in the network pattern between WM manipulation and maintenance, as described below.

#### 2.2.3 Construction of directional CFC networks

Directional functional connectivity between electrode pairs was quantified using the MI. It is worth noting that “directional” here follows the conventional definition of phase-amplitude coupling (Canolty & Knight, 2010; Tort et al., 2010), referring to how the low-frequency phase statistically modulates the high-frequency amplitude across sites, rather than implying information transmission. The connection from a source electrode (A) to a target electrode (B) was then defined as the MI quantifying the cross-frequency coupling between the low-frequency band of A and the high-frequency band of B.

To extract the network’s significant directional architecture, we identified pairs of electrodes exhibiting statistically asymmetric coupling. For all _32_C^2^ = 496 possible pairs, we computed a directed MI contrast (ΔMI = MI(A→B) – MI(B→A)) for each participant. A one-sample test against zero determined the significance of ΔMI for each pair across the cohort. The resulting p-values (0.05) were corrected for multiple comparisons using the Holm-Bonferroni method. This statistical filtering produced a directed network graph where edges represent significant cross-electrode modulation. This network was constructed separately for the maintenance and manipulation tasks.

#### 2.2.4 Performance evaluation of cognitive tasks

The tasks in this project were designed to generate relatively high accuracy in participants’ performance. As such, the behavioral performance indicator was primarily assessed using RT (speed). This design choice aimed to reduce the dimensionality of behavioral variability by focusing on RT alone, minimizing confounding from a trade-off between speed and accuracy. It is noted that high accuracy in this context does not imply a ceiling effect, as RT still reflects meaningful differences in task demands. Participants whose accuracy in both WM maintenance and manipulation tasks was below 80% (3 out of 68 participants) were excluded from the behavioral analysis. The average accuracies for the maintenance and manipulation WM tasks were 93.4% and 90.2%, respectively.

To construct latent variables for WM maintenance, WM manipulation, sensorimotor speed, and operation span, trials in each task were divided into equal partitions, and the median RTs within each partition were calculated. Specifically, the WM maintenance, manipulation, and sensorimotor speed tasks were divided into four equal partitions, each consisting of 10 trials organized according to trial order. For the operation span task, trials were divided into five partitions, each corresponding to one of the five set sizes. With three trials per set size, we calculated the median RT for each set size as an indicator of the participants’ performance across different levels of cognitive load.

#### 2.2.5 Factor analysis and structural equation modeling

The factor analysis was conducted with two primary objectives: (1) to evaluate the reliability of the newly designed task paradigm and (2) to compare WM maintenance and manipulation from both behavioral and neural indicators.

Confirmatory factor analysis (CFA) was used to compare the fit of two distinct models: one incorporating both maintenance and manipulation as separate factors, and another featuring a single, unified factor. A chi-square difference test was employed to evaluate the relative fit of the two models. In addition to the comparison of model fit between the one-factor and two-factor models using CFA, we further conducted exploratory factor analysis (EFA) to examine whether the loading patterns of different indicators across maintenance and manipulation tasks exhibited a structured pattern consistent with the separability of the two constructs.

Furthermore, structural equation modeling (SEM) was employed to investigate the relationships between the factors of sensorimotor speed, WM maintenance and manipulation derived from our tasks, and WM ability from the operation span task. In SEM, regressions were constructed between sensorimotor speed and the two WM factors, respectively, to assess the direct contribution of sensorimotor speed to maintenance and manipulation. The difference in the path coefficient (e.g., sensorimotor ability contributes more to maintenance and less to manipulation) would provide additional characteristics contrasting the two WM components. The relationships between the factor of WM ability from the operation span task and all other factors were evaluated by correlation at the latent variable level. The reliability of each factor is reflected by the magnitude and consistency of factor loadings across the separate indicators.

All the analyses above were conducted on the neural indicators of MI in an identical way. Finally, SEM analysis was conducted to assess the extent to which the neural correlates of WM (i.e., CFC) predicted the behavioral factors of WM abilities.

## 3 Results

### 3.1 Behavioral performance in WM maintenance and manipulation tasks

**Fig. 2.**
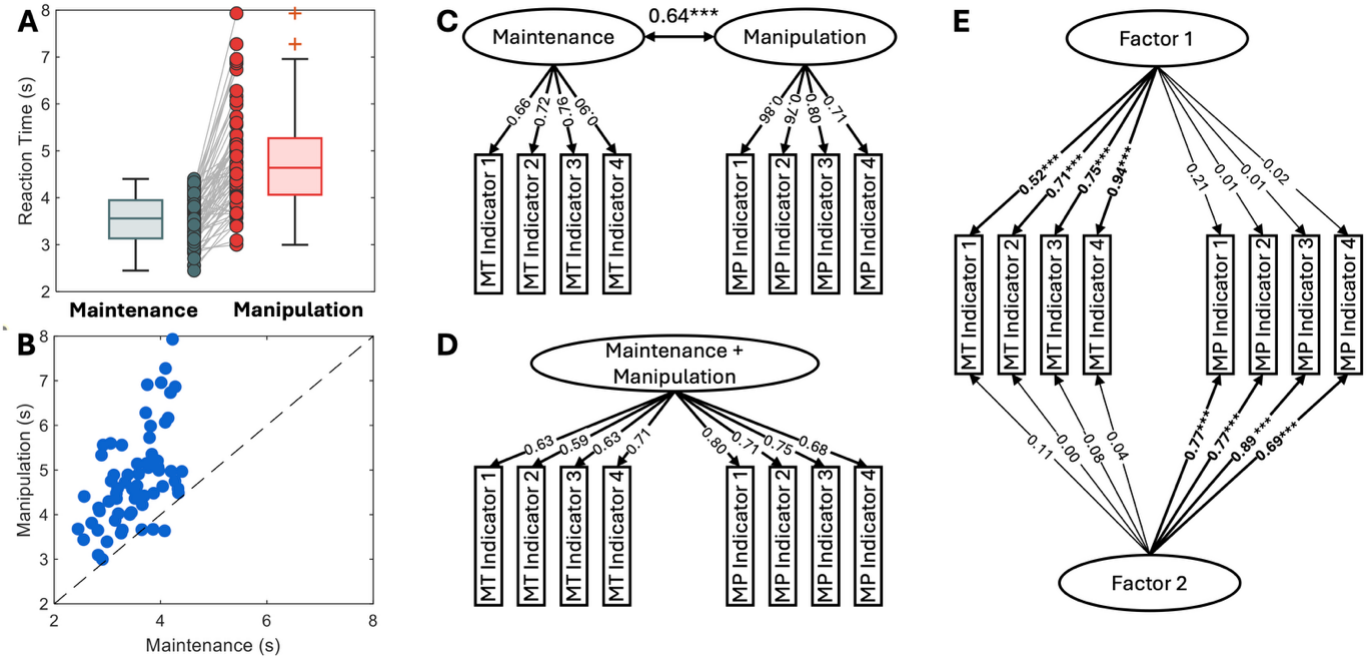
Results characterizing the differences in behavioral variability between WM maintenance (MT) and manipulation (MP) tasks. (A) Box plot showing average RT for both the WM maintenance and manipulation tasks, with individual data points plotted for each participant in both conditions. (B) Scatter plot showing the relationship between participants’ average RTs in WM maintenance and manipulation tasks. (C) CFA results for a two-factor model distinguishing maintenance and manipulation. (D) CFA results for a one-factor model treating WM maintenance and manipulation as a unified construct. (E) EFA results revealing two distinct underlying factors within the WM data. Asterisks indicate significance levels: ***: p < 0.001; **: p < 0.01; *: p < 0.05. Asterisks mark significant indicator loadings in the EFA (panel E) and the covariance between factors in the CFA (panel C). All CFA loadings (panels C–D) were significant at p < 0.001 and are unmarked by asterisks for cleanliness in the figure.

The behavioral results showed a clear distinction between WM maintenance and manipulation tasks in RT (Fig. 2A). RTs were significantly longer in the WM manipulation task than in the WM maintenance task, with a mean difference of 1.27 seconds. A paired t-test confirmed a statistically significant difference between the two conditions (p < 0.001, Cohen’s d = 1.46), as expected given the greater cognitive demands imposed by WM manipulation. Accuracy was high in both tasks (maintenance: 37.34 ± 2.60 out of 40; manipulation: 36.09 ± 3.58 out of 40), with only a small difference between them (Cohen’s d = 0.36). Compared with the large effect size in the RT difference (Cohen’s d = 1.46), the smaller accuracy effect indicates that the behavioral difference was predominantly localized in RT, which is in line with the task design. The RTs from the two tasks showed a moderate correlation (r = 0.55, p < 0.001) (see Fig. 2B).

Both CFA and EFA results indicated differential behavioral variability between the WM maintenance and manipulation tasks. For CFA, two competing models were built: a two-factor model (Fig. 2C), which treats WM maintenance and manipulation as separate constructs modeled by different factors, and a one-factor model (Fig. 2D), which conceptualizes both processes as a single construct and uses a single factor to explain the variability across all indicators. The two-factor model showed a significantly better fit, as indicated by the chi-square difference test (Δχ²(1) = 52.07, p < 0.001). Model fit indices for the two-factor model were: χ²(19) = 18.00, p = 0.52, RMSEA = 0.00, 90% CI [0.00, 0.10], CFI = 1.00, TLI = 1.01, SRMR = 0.04, AIC = 1236.69, BIC = 1290.67; and for the one-factor model: χ²(20) = 70.07, p < 0.001, RMSEA = 0.20, 90% CI [0.15, 0.25], CFI = 0.80, TLI = 0.72, SRMR = 0.09, AIC = 1286.76, BIC = 1338.58. This superior fit was replicated in split-half analysis (half 1: Δχ²(1) = 18.96, p < 0.001; half 2: Δχ²(1) = 35.84, p < 0.001). EFA also revealed a clear two-factor solution (Fig. 2E), with indicators from the WM maintenance task loading significantly onto one factor (p < 0.001), and those from the WM manipulation task loading significantly onto the other (p < 0.001). Across both CFA and EFA, split-half analyses yielded consistent two-factor structures, with consistent loading patterns and inter-factor covariances across the two halves of the same sample, supporting the stability and robustness of the model. Detailed model-fit indices and parameter estimates for the split-half analysis are provided in the Supplementary Materials (Tables S1 and S2).

### 3.2 Structural equation modeling of behavioral data

**Fig. 3.**
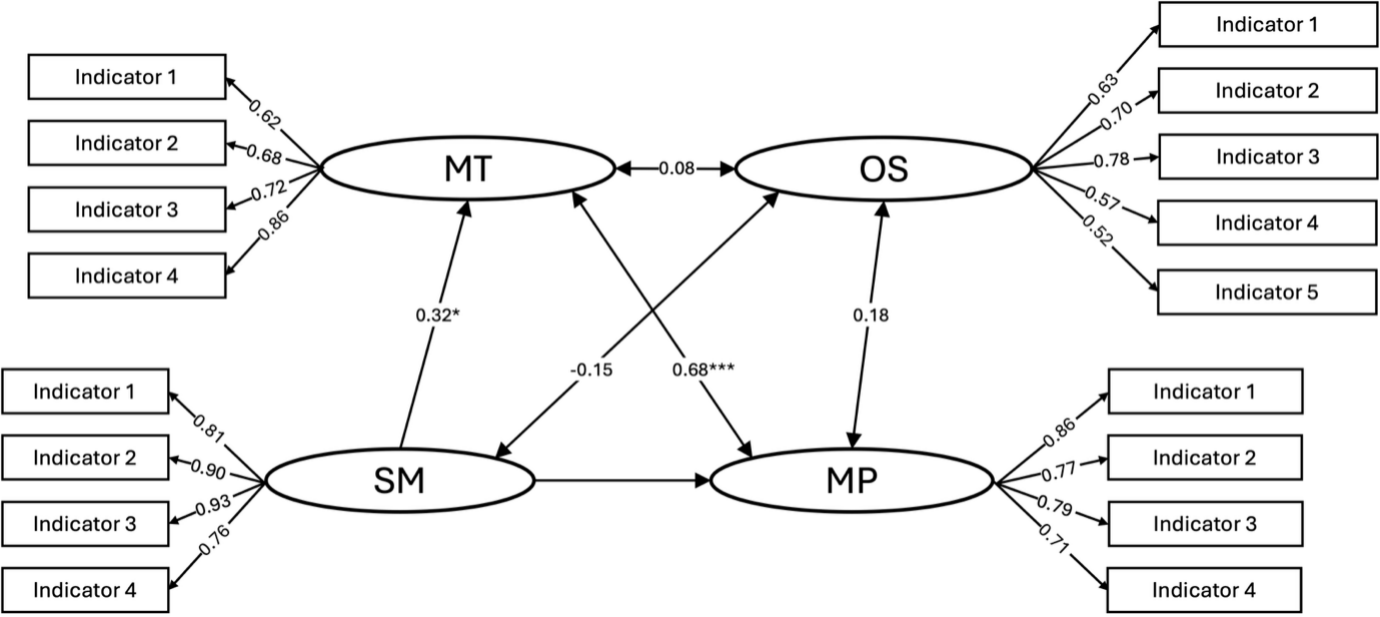
Structural equation modeling analysis illustrating the relationships among factors of behavioral performance for WM maintenance (MT), WM manipulation (MP), sensorimotor speed (SM), and operation span (OS). Asterisks indicate significance levels: ***: p < 0.001; **: p < 0.01; *: p < 0.05 for the covariances and regression coefficients shown in the model. All factor loadings were significant at p < 0.001 and are unmarked for cleanliness in the figure.

To examine the structural relationships between WM maintenance, WM manipulation, sensorimotor speed, and operation span task performance, SEM was conducted, as depicted in Figure 3. Model fitting results: χ²(113) = 127.65, p = 0.16, RMSEA = 0.05, 90% CI [0.00, 0.08], CFI = 0.97, TLI = 0.97, SRMR = 0.06, AIC = 2643.10, BIC = 2766.16.

The SEM revealed a strong covariance between WM maintenance and WM manipulation (0.68, p < 0.001), indicating that the two tasks share underlying cognitive processes. Sensorimotor speed exhibited a significant regression coefficient with WM maintenance (0.32, p < 0.05), but a much weaker and non-significant coefficient with WM manipulation (-0.06, p = 0.68). These results suggest that sensorimotor speed is strongly associated with WM maintenance performance but not with WM manipulation, in line with the assumption that the manipulation task entails more complex cognitive processes. Note that the current sample size may be relatively low for a typical SEM analysis. In order to validate if the relationship between different factors is a stable result, we conducted a supplementary path analysis using observed variables. The results from the path analysis showed a similar pattern of internal relationships between the main variables (see Supplementary Table S3).

The latent factor representing the operation span task showed relatively low covariances with both WM maintenance (0.08) and WM manipulation (0.18), suggesting a high heterogeneity in the cognitive processes engaged in the operation span task and the two WM tasks in this study. The covariance between the operation span factor and sensorimotor speed was also weak and non-significant (-0.15). Split-half analyses showed consistent factor loadings and covariances across halves, indicating a stable model structure (see Supplementary Table S4).

### 3.3 Difference in neural cross-frequency coupling between WM maintenance and manipulation

**Fig. 4.**
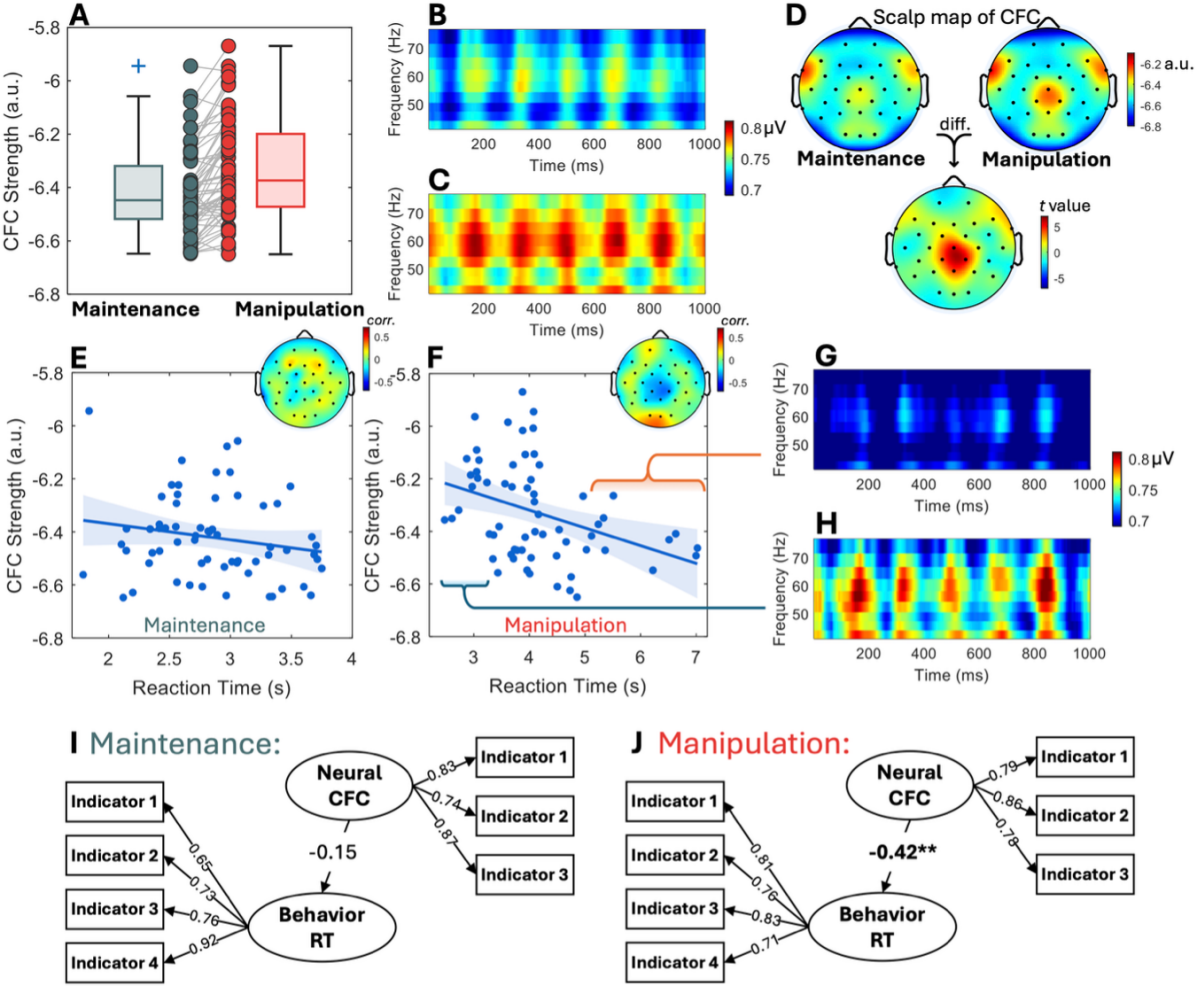
Neural results showing the distinction between WM maintenance and manipulation. (A) Box plot showing MI for WM maintenance and manipulation tasks, with individual data points for each participant. (B, C) Visualized CFC pattern during the task under WM maintenance and manipulation conditions (x-axis: milliseconds). This plot is the averaged time-shifted time-frequency representations across all 1-second segments (see Section 2). (D) Topographical maps of MI for each condition, and corresponding t-values indicating MI differences between maintenance and manipulation. (E, F) Scatter plot depicting the relationship between MI and RT in the WM maintenance task (E) and the WM manipulation task (F), with regression lines and 95% confidence intervals. Inset topographical maps show electrode-wise correlations between RT and MI. (G, H) Visualized CFC for the slowest RT group (10 participants with the slowest RT) and the fastest RT group (10 participants with the fastest RT). (I, J) SEM results for RT and MI in WM maintenance (I) and manipulation (J) tasks, respectively. Asterisks indicate significance levels: ***: p < 0.001; **: p < 0.01; *: p < 0.05 for the regression coefficients shown in the model. All factor loadings were significant at p < 0.001 and are unmarked for cleanliness in the figure.

Consistent with the behavioral findings, the MI, which indexes phase-amplitude CFC, was consistently higher in the WM manipulation task compared to the WM maintenance task across participants (Fig. 4A). The values in Figure 4A were calculated as the average MI across central-parietal electrodes (CP1, CP2, Pz and Cz), where the task-related CFC differences between the WM maintenance and manipulation conditions were the most pronounced (see Fig. 4D). Note that here we refer to the average MI across the electrodes of interest as “CFC strength”. A paired t-test confirmed a significant difference between the two (p < 0.001; see Fig. 4A). Figure 4B and C descriptively show the difference in the magnitude of CFC between tasks. The topographical maps in Figure 4D show the spatial pattern of this task-related CFC difference across the scalp.

In addition to the average magnitude difference in MI, cross-individual variability in MI paralleled behavioral data, with a two-factor model providing a better fit than a single-factor model based on chi-square test (p = 0.019). The test used MI from the Cz electrode, located at the center of the region showing the strongest task-related CFC difference.

The relationship between CFC (measured by MI) and behavioral performance across individuals is shown by the scatter plots in Figure 4E and F. MI was more strongly correlated with RT in the manipulation task (r = -0.38, p = 0.002) than in the maintenance task (r = -0.14, p = 0.28), suggesting that MI more reliably reflects task performance during manipulation. This stronger association indicates that CFC is more closely associated with information manipulation than maintenance. To further visualize this relationship, participants were divided into two groups based on median RT in the manipulation task: the 10 participants with the fastest RTs and the 10 with the slowest RTs. Phase-amplitude CFC visualizations for these two groups showed that the slower group in the manipulation task exhibited less pronounced CFC (see Fig. 4G, H).

The topographical maps of the correlation between RT and MI again revealed that relevant neural correlates were predominantly observed in the central-parietal region in the manipulation task (see Fig. 4E, F). This region overlaps with the area showing significant MI differences between maintenance and manipulation, thus providing converging neural evidence for the differentiability of the underlying constructs.

SEM was conducted to assess the extent to which CFC strength (defined as MI averaged across CP1, CP2, Pz, and Cz) predicted corresponding RTs in both tasks. MI in the maintenance task showed a non-significant predictive relationship with maintenance RT (path coefficient = -0.15, p = 0.30), while MI in the manipulation task strongly predicted manipulation RT (path coefficient = -0.42, p < 0.01) (see Fig. 4I, J), which is consistent with the scatter plots. In summary, MI predicts behavioral performance, but only in the manipulation task. Model fitting results for the maintenance model: χ²(13) = 12.68, p = 0.47, RMSEA = 0.00, 90% CI [0.00, 0.12], CFI = 1.00, TLI = 1.00, SRMR = 0.06, AIC = 1082.19, BIC = 1129.34. For the manipulation model: χ²(13) = 15.50, p = 0.28, RMSEA = 0.06, 90% CI [0.00, 0.14], CFI = 0.99, TLI = 0.98, SRMR = 0.05, AIC = 1022.91, BIC = 1070.06. Split-half analyses of these SEMs showed consistent parameter estimates across halves, indicating stable neural-behavioral associations (see Supplementary Table S5).

### 3.4 Difference in the functional networks between WM maintenance and manipulation revealed by CFC

To better understand how WM maintenance and manipulation differ in their underlying neural substrates qualitatively rather than merely quantitatively, we examined the associated functional network patterns. Here, cross-electrode MI was used to represent functional connectivity between two electrodes with directionality (see Section 2).

**Fig. 5.**
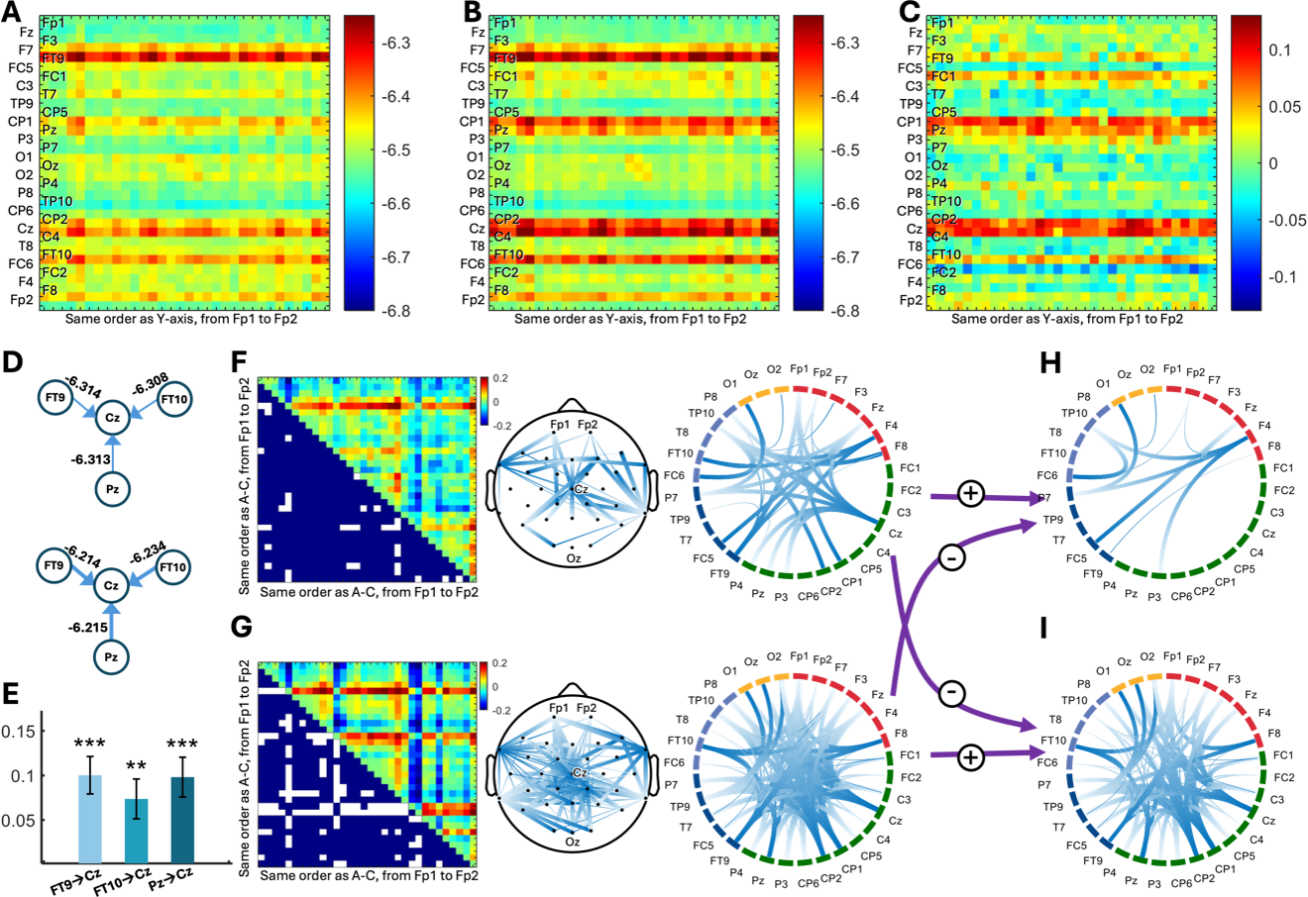
Task-related differences in cross-electrode modulation indices and connectivity patterns. (A–C) Whole-scalp cross-electrode MI matrices for the maintenance task (A), the manipulation task (B), and their difference matrix (C; manipulation minus maintenance). (D) Schematic illustration of the three electrodes (FT9, FT10, and Pz) that exhibited the strongest modulation to Cz, shown separately for the maintenance (top) and manipulation (bottom) tasks. (E) Bar plots showing the strength of modulation from FT9 → Cz, FT10 → Cz, and Pz → Cz. All three connections showed significantly stronger modulation in the manipulation condition (p < 0.01). (F, G) Directionality results for maintenance (F) and manipulation (G). In each case, the left panel shows the directionality matrices: the upper triangles display the directional MI values, and the lower triangles highlight electrode pairs with significant task-related differences (white spots; paired t-tests, p < 0.05). Directionality values were calculated as MI(A→B) − MI(B→A), where A→B denotes the low-frequency phase of electrode A modulating the high-frequency amplitude of electrode B. The middle and right panels show the corresponding significant directionality networks for each task, with line width representing the strength of modulation. Dark blue indicates target electrodes (modulated), and light blue indicates source electrodes (modulating). (H, I) Connections significant exclusively in the maintenance task (H) or in the manipulation task (I).

We first computed whole-scalp connectivity matrices. MI matrices were computed separately for the maintenance (Fig. 5A) and manipulation (Fig. 5B) tasks, along with their difference matrix (Fig. 5C). The results indicate that most electrodes across the scalp exhibit modulation effects on Cz, suggesting that low-frequency activity from multiple regions modulates high-frequency activity at Cz.

From the MI matrices, we identified three electrodes that exhibited the strongest modulation to Cz: FT9, FT10, and Pz. We then selected these three electrodes to demonstrate that the difference in CFC between maintenance and manipulation occurs not only within electrodes but also across electrodes. The strength of their modulation to Cz is visualized in Figure 5D for the two tasks. The t-tests showed that the modulation from each of these electrodes to Cz (FT9 → Cz, FT10 → Cz, and Pz → Cz) was significantly stronger in the manipulation task than in the maintenance task (Fig. 5E; ts > 3.30, ps < 0.01; all remained significant after correction for multiple comparisons by the Holm-Bonferroni method). These results show that the manipulation not only engages stronger within-electrode CFC at Cz (Fig. 4) but also enhances cross-electrode, cross-region modulations.

To explore whether the task type (maintenance or manipulation) alters the functional network in ways that go beyond simply increasing modulation strength, we analyzed the directionality patterns of the network. Directionality refers to asymmetrical modulations between electrode pairs, calculated as MI in one direction minus MI in the opposite direction. A larger difference indicates greater asymmetrical modulation between two electrodes, while a smaller difference suggests more comparable reciprocal modulation. Here, modulation refers to low-frequency activity at one electrode modulating high-frequency activity at another.

If manipulation alters the functional network in a qualitatively distinct way, this should be reflected in the pattern of directional modulation between electrode pairs. The directionality matrices for the maintenance and manipulation conditions are shown in the upper triangles of Figure 5F and G (left panels, respectively). To statistically evaluate task-related differences, we performed paired t-tests on directionality values between conditions, identifying significantly different electrode pairs (p < 0.05, Holm-Bonferroni corrected). These pairs are represented by white spots in the lower triangles of Figure 5F and G (left panels). The full significant directionality networks for the maintenance and manipulation tasks are shown in the middle and right panels of Figure 5F and G, respectively. Here, dark blue indicates target electrodes (modulated), light blue indicates source electrodes (modulating), and line width reflects the strength of modulation. The manipulation task elicited a substantially more extensive network.

To further demonstrate that the two WM components engage distinct modulation networks, we identified connections that were significant in one task but not the other. Figure 5H shows links exclusive to the maintenance task, while Figure 5I shows those exclusive to the manipulation task. This analysis revealed an extensive, manipulation-specific network (Fig. 5I). Although the maintenance-specific network was more sporadic (Fig. 5H), its survival after multiple comparisons correction confirms that maintenance also involves a unique network. In summary, the network analysis reveals distinct cross-region interactions for each WM component, with manipulation recruiting a profoundly different and more complex network dynamic, indicating a qualitative difference between the two processes.

### 3.5 Validation analyses

To assess the stability of the reported CFC effects, we further conducted a set of validation analyses examining potential dependencies on analytical and task-related factors. These analyses evaluated (a) the validity of frequency ranges used to compute the MI, (b) whether the observed CFC patterns were critically influenced by motor-related activity during the response period, and (c) whether the stronger CFC observed during WM manipulation merely reflected magnitude increase or involved structural change in cross-individual variability.

#### 3.5.1 Validity of the frequency band selected

**Fig. 6.**
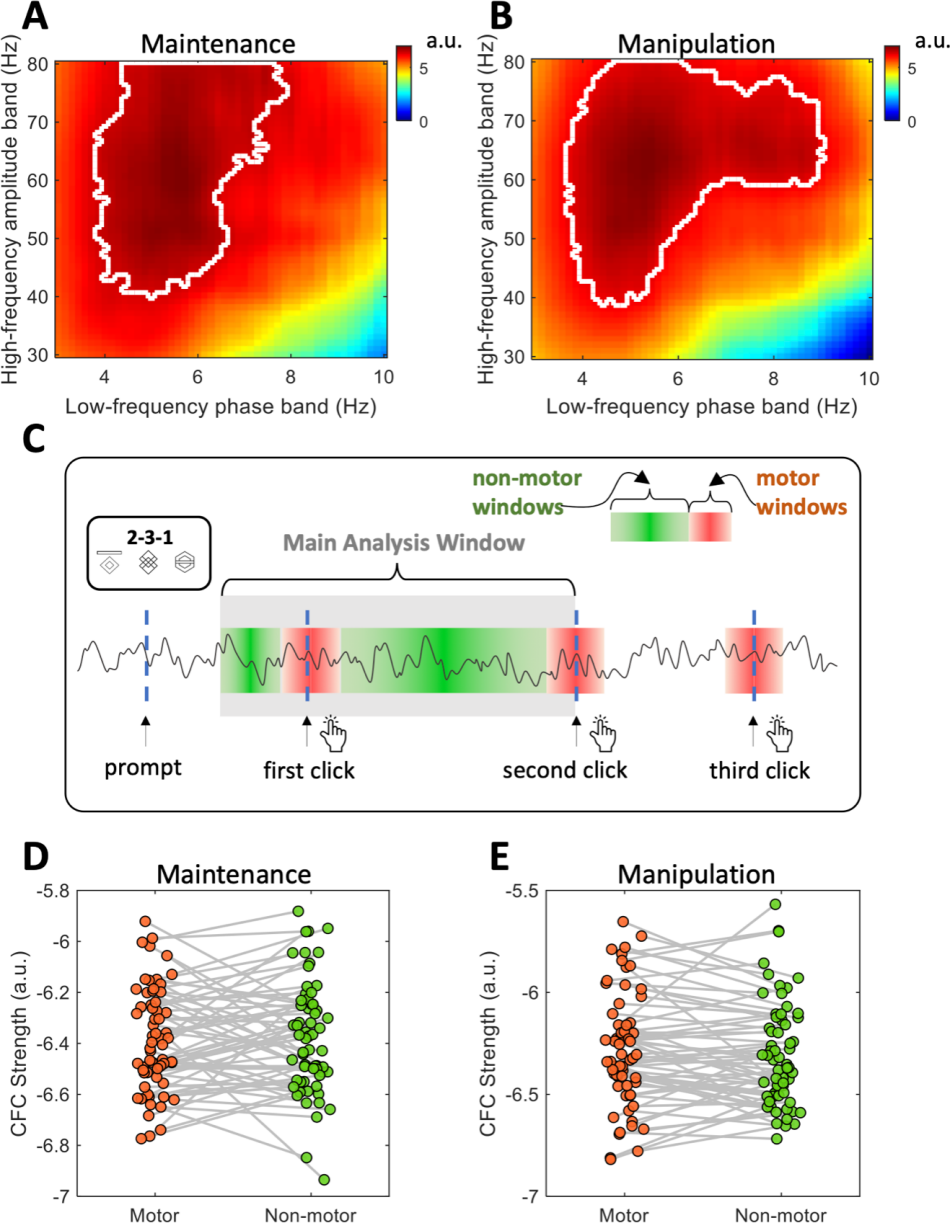
Validation analyses. (A, B) MI distributions across pairs of low-frequency phase and high-frequency amplitude bands for the WM maintenance (A) and WM manipulation (B) conditions. The white contour marks phase-amplitude frequency combinations exceeding a permutation-based reference level (null mean + 3 SD). (C) Illustration of temporal segmentation used to identify motor and non-motor epochs. The gray zone marks the main analysis window (500 ms after prompt to the second click); red and green areas indicate motor (±200 ms around clicks) and non-motor windows, respectively. (D, E) Individual MI values comparing motor and non-motor segments for WM maintenance (D) and WM manipulation (E). No significant differences were observed between motor and non-motor epochs in either condition.

To ensure that the observed CFC was not a specific result from carefully selected combinations of low- and high-frequency bands, we recomputed the CFC strength (MI) across a fine-grained matrix of frequency combinations at the Cz electrode (Fig. 6A, B).

Low-frequency phase was obtained from 3 to 10 Hz with a 1-Hz bandwidth, and high-frequency amplitude was obtained from 30 to 80 Hz with a 10-Hz bandwidth. Each pair of phase and amplitude was used to calculate MI following the same procedure described earlier.

Figure 6 shows that both WM maintenance and manipulation conditions exhibited a coherent cluster of elevated MI values centered around 4–8 Hz (phase) and 40–80 Hz (amplitude). Outside this range, MI values were consistently lower and spatially diffuse, indicating that the theta-gamma frequency ranges used capture the dominant effect of phase–amplitude interaction in this dataset. To help interpret the color scale, we overlaid a white contour indicating phase-amplitude frequency combinations where the group-average MI exceeded a permutation-based null reference, constructed by permuting MI values across the phase-amplitude frequency matrix within participants. The null reference was defined as the mean of the permutation-derived null distribution plus three SD. These results demonstrate that the observed effects are not artifacts of parameter selection but reflect a systematic pattern of intrinsic neural dynamics underlying WM processing.

#### 3.5.2 Examining the motor dependency of CFC

The time window used for calculating CFC involves the main period of WM processing, but this time window also inevitably contains motor activity. To examine whether the motor activity within this window critically influenced CFC strength, we compared MI values from motor-related epochs (200 ms before and 200 ms after each click) with those from non-motor intervals (Fig. 6C). Figure 6C illustrates the time windows used for this comparison, with the gray zone marking the main analysis window for CFC and red/green zones indicating motor and non-motor time windows, respectively.

No significant MI differences were found between motor and non-motor segments in either condition (*maintenance*: *t*(62) = -0.46, p = 0.65, 95% CI [–0.064, 0.040]; *manipulation: t*(62) = 0.88, p = 0.38, 95% CI [–0.026, 0.067]). These results suggest that the CFC strength reported in the main analysis is unlikely to be dominated by transient activity associated with motor preparation or execution, although subtle motor contributions cannot be fully ruled out.

In addition, to address the possibility that residual motor activity may persist beyond brief motor-locked windows, we conducted a complementary control analysis comparing MI values between the prompt-to-first-click and first-to-second-click intervals, which both involve active working memory processing but differ theoretically in their motor-related demands. No significant differences were observed in either condition.

#### 3.5.3 Intra-task and inter-task analyses of individual variability in CFC

SEM in Section 3.3 showed that WM maintenance and manipulation differ not only in mean CFC strength but also in the structure of cross-individual variability. To ensure that this differentiation was not simply driven by differences in overall MI magnitude, we further examined intra-task stability in CFC.

For each participant, all the trials were divided into lower- and higher-MI magnitude subsets, and model fits were compared between a one-factor model (treating all MI indices as reflecting a single factor) and a two-factor model (separating low- and high-MI subsets). In both the maintenance and manipulation conditions, the difference between one- and two-factor models was non-significant (p = 0.33 and p = 0.52, respectively), indicating that variations in MI magnitude within a task do not change the underlying latent structure of individual differences.

By contrast, as shown earlier, the comparison between maintenance and manipulation showed a significantly better fit for the two-factor model (p = 0.019). These results confirm that the stronger theta-gamma coupling observed during WM manipulation reflects a qualitatively distinct inter-task structure of individual differences rather than an effect of generalized task difficulty or overall CFC strength. Detailed model structures and fit indices are provided in Supplementary Figure S1 and Table S6.

## 4 Discussion

### 4.1 Summary

The current study aimed to design new task paradigms to advance the understanding of the WM constructs by comparing WM maintenance and manipulation while deliberately minimizing the influences of other factors, such as task-specific strategies, processes, skills, domain knowledge, and the interaction between speed and accuracy in behavioral measures. Crucially, the study compared WM maintenance and manipulation in both behavioral and neural data.

The psychometric properties revealed two homogeneous yet dissociable factors capturing the cross-individual variability in task performance, supporting that WM maintenance and manipulation components are separable (Baddeley, 1996, 2000; D’Esposito & Postle, 2015; D’Esposito et al., 1999; Glahn et al., 2002; Masse et al., 2019; Smith & Jonides, 1999; Wager & Smith, 2003). The factor analysis employed here, however, has an inherent limitation in capturing task content-specific variabilities, which will be discussed below. Beyond the behavioral data, neural evidence also indicated the difference between the two WM components: 1) Elevated phase-amplitude CFC and structured changes in cross-individual variability during the manipulation task suggest the engagement of more intense and distinct neural computational processes. 2) The differential association between CFC strength and behavioral performance across tasks highlights their distinct reliance on CFC-mediated neural computation. 3) WM manipulation recruited a different functional connectivity network from WM maintenance.

### 4.2 Different behavioral and neural variability associated with WM maintenance and manipulation

The WM construct has been conceptually divided into the functional modules of information maintenance and manipulation in extensive discourses of cognitive research (Aben et al., 2012; Baddeley, 1996, 2000; D’Esposito et al., 1999; Eriksson et al., 2015; Glahn et al., 2002; Masse et al., 2019; Vergauwe et al., 2014). Although prior studies have examined behavioral differences between the two abilities, these comparisons typically relied on tasks that differed substantially in design and content (Engle et al., 1999; Swanson & Kim, 2007). In this study, we used a unified set of two tasks that only differ in the involvement of a manipulation process to examine the separability of the two sub-constructs. The distinction between WM maintenance and manipulation in this study is evidenced through multiple findings supporting that the two functional modules are separable within the WM system.

First, the chi-square difference test of the cross-individual behavioral variability supports a two-factor model that treats WM maintenance and manipulation as distinct factors, showing a significantly better fit than a single-factor model that assumes their homogeneity. This indicates that the variability in behavioral performance in the WM manipulation task is statistically distinct from the variability in the WM maintenance task, suggesting different sources underlying these variabilities. An EFA further corroborated this finding. The EFA automatically searched for independent projections across eight performance indicators, without knowledge of which task each indicator originated from. The fact that EFA converged to the result of two loading distributions clearly and exclusively absorbed into each of the two tasks indicated strongly distinct patterns of variability. Consistent with these behavioral findings, neural indicators (CFC measured by MI values) also support the two-factor model by replicating the psychometric characteristics.

A potential concern at this point is that the manipulation task simply involves an additional operational step, resulting in longer RT. However, an increase in RT does not necessarily imply the engagement of a distinct cognitive construct. The added step could merely prolong the same underlying process without altering its nature. For example, requiring a participant to press a button twice instead of once would increase task duration without introducing a qualitatively new operation. Another concern about whether the differential variabilities imply different underlying cognitive constructs lies in the limitation from task differences. Although we have explicitly noted this limitation (Friedman & Miyake, 2017) and designed our tasks within a unified framework that only differs in the involvement of an additional manipulation step, it is theoretically possible that the two tasks may elicit different cognitive processes resulting in factor dissociation in a way that is not directly related to the different nature of maintenance and manipulation. Concerning these limitations, we provided additional neural evidence supporting the difference between the two WM components.

The manipulation task elicited elevated MI values on average (see Fig. 4A), indicating stronger phase-amplitude coupling than in the maintenance condition. This elevation cannot be attributed to trial duration, as CFC is computed as an intrinsic feature of the EEG signal and is not affected by its length (see Section 2). The increased MI reflects qualitatively distinct neural dynamics, characterized by heightened computation, coordination, and integration, rather than simply a longer version of the same process. This aligns with theoretical models proposing that manipulation imposes greater cognitive demands than maintenance (Aben et al., 2012; Crone et al., 2006; D’Esposito et al., 1999), reinforcing the interpretation that WM manipulation and maintenance are functionally separable processes rooted in distinct neural mechanisms.

The predictive relationship between CFC and behavioral performance provides an additional layer of evidence. CFC showed a strong predictive relationship with RT in the manipulation task, consistent with the cognitive demands of actively processing information. In contrast, the weaker, non-significant association between CFC and RT in the maintenance task suggests that CFC is less relevant to performance in that condition, further supporting the distinction between the neural processes underlying manipulation and maintenance.

To situate these behavioral and neural distinctions within the broader literature, it is important to note that a large body of neuroimaging research has consistently identified distinct neural signatures for WM maintenance and manipulation. Functional MRI and EEG studies have repeatedly shown greater activation and frontoparietal connectivity during manipulation tasks, reflecting increased executive control and dynamic updating demands (Berger et al., 2019; D’Esposito et al., 1999; Wager & Smith, 2003). The reordering paradigm employed here parallels previous designs used to isolate manipulation-specific processes, such as serial-reordering and letter-sequencing tasks (D’Esposito et al., 1999; Jablonska et al., 2020; Owen et al., 2005; Van Hecke et al., 2010; Veltman et al., 2003). Building on these well-established paradigms and findings, the present study contributes complementary evidence by focusing on the temporal dynamics of neural coordination rather than spatial activation patterns. While much of the previous work emphasized group-level activation contrasts, relatively few studies have examined cross-individual variability or the oscillatory interactions that organize such processes over time. By integrating this established paradigm with electrophysiological analysis of CFC, the present study extends this literature by characterizing the temporal neural mechanisms that differentiate manipulation from maintenance.

It is worth noting that the two neural factors show a high latent correlation (r = 0.94), indicating a large amount of shared variance between maintenance and manipulation in the CFC measure (MI) as compared to behavioral data (r = 0.64). This is possibly due to the fact that MI is a neural signal feature derived from the statistics of basic features, including amplitudes and phases of band-specific oscillations, which possesses high in-session stability as compared to behavioral measures. Nevertheless, the statistical difference between the variability of maintenance and manipulation is still noticeable in the factor analysis that favors their distinction, which is contrasted with the high correlation (1 and 0.995) obtained in the mixed-and-split control analysis (Supplementary Fig. S1).

### 4.3 The hierarchical cognitive structure revealed by the relationships among different tasks

The relationships among sensorimotor speed, WM maintenance, and WM manipulation shown by the SEM (Fig. 3) revealed a sensible hierarchical structure from a cognitive system point of view. In this hierarchical system, sensorimotor speed serves as the foundational layer, reflecting the ability to process stimulus and respond efficiently. On top of this foundation, WM maintenance incorporates not only the basic sensorimotor processes but also the process of retaining information over a brief period. This added layer of cognitive demand makes WM maintenance more complex than basic sensorimotor processing. WM manipulation, in turn, builds upon WM maintenance by introducing additional cognitive processes, such as restructuring, updating, and transforming information. Along with this hierarchical increment, each successive layer depends on the preceding one while adding new functional demands that require distinct mechanisms. Consistent with this architecture, behavioral variability in the sensorimotor speed task contributes noticeably to variability in the WM maintenance task, but far less to the manipulation task. This prediction is fully in line with what SEM shows (Fig. 3). The manipulation task does not merely extend maintenance—it engages additional cognitive processes that generate greater variability, thereby weakening its association with sensorimotor speed and differentiating it further from WM maintenance.

Findings from the operation span task offer additional perspective on this hierarchical structure. Unlike the WM maintenance and manipulation tasks designed in the current study, the operation span task, which is a type of complex span task, combines memory recall with mental calculation. Other complex span tasks, like reading span, also involve more cognitive processes such as reading comprehension. This added complexity in task processing likely introduces a significant amount of heterogeneous variability into the behavioral indicator, which may explain two results observed in the current study. The first result is its low correlations with both WM maintenance and manipulation. This suggests that while the operation span task is commonly used to assess the WM construct, its composite nature might introduce significant variability in addition to the central constructs of WM maintenance and manipulation. The second result is the lower and more variable factor loadings across the indicators of the operation span task—again, likely due to the heterogeneous nature of the task.

Notably, SEM models based on operation span task accuracy did not converge reliably, necessitating the use of RT from the operation span task to analyze the psychometric characteristics and its correlation with other constructs. This may reflect the diverse sources of variability affecting accuracy, such as anxiety, attention lapses, differences in task difficulty between set sizes, or complexity in the response format (here, selecting and clicking from a large matrix of letters) could all contribute to the accuracy.

Together, these findings suggest the necessity of re-evaluating the effect of the complexity of WM-related tasks on measurement. The low correlations between the two newly designed WM tasks and the operation span task indicate that tasks with more composite demands may reflect a broader range of cognitive processes. This calls for the development of tasks that more selectively capture variability in targeted cognitive functions. The two WM tasks presented in this study represented an effort in this direction. However, limitations of the current tasks remain and will be discussed further below.

### 4.4 CFC reveals the differences between WM manipulation and maintenance

The observed relationship between CFC and WM tasks in this study contributes to existing research on CFC in cognitive processing by highlighting its differential involvement in WM manipulation and maintenance. Spontaneous CFC activity has been widely found to be associated with various complex cognitive processes, revolving around central information processing (Canolty & Knight, 2010; Jirsa & Müller, 2013; Miller et al., 2018; Tamura et al., 2017). These studies with convergent findings have largely confirmed that CFC plays a role in basic neural computation underlying cognitive functions. The present study adds two new contributions to this literature. First, it provides a more detailed role of CFC in the neural computational system: CFC is more strongly associated with information manipulation than with simple maintenance. Second, it provides novel evidence that CFC can index individual differences in central information processing ability.

Theoretically, theta-gamma CFC has been widely discussed as a key neural code underlying the organization and maintenance of information in WM (Lisman & Idiart, 1995; Lisman & Jensen, 2013). In this framework, slower theta oscillations provide a temporal scaffold that organizes the timing of faster gamma bursts representing clustered computation. This hierarchical nesting is proposed to enable multiple items to be sequentially represented, supporting the structured maintenance of ordered information (Bahramisharif et al., 2018; Sauseng et al., 2019). Empirical studies using intracranial and scalp EEG have demonstrated load-dependent theta-gamma coupling in the hippocampus and cortical regions during WM retention (Axmacher et al., 2010; Holz et al., 2010), as well as enhanced coupling strength for correctly maintained items (Jones et al., 2020). These findings establish theta-gamma coupling as a reliable neural signature of the temporal coordination supporting WM operations. Within this framework, gamma-band activities are thought to be associated with individual memory items, while slower theta rhythms are associated with temporal structuring (Roux & Uhlhaas, 2014).

In line with this framework, information maintenance and manipulation involve differentiated functional processes that may be manifested in CFC. Maintenance has been commonly referred to as the process of holding information in an accessible state with stability over a certain period, which mainly relies on neural mechanisms that support the continuous representation of information (Axmacher et al., 2007; Miller & Cohen, 2001; Roberts et al., 2013; Shi & Yu, 2024). The maintenance processes are more representational in nature. In contrast, manipulation mainly involves active processing of the maintained information, such as integration, filtering, transformation, updating, or restructuring depending on task demands, which is more computational in nature. Therefore, tasks recruiting different amounts of maintenance and manipulation would presumably exhibit differential CFC dynamics. First, the stronger CFC strength observed during manipulation may reflect higher demand in coordination supporting the flexible transformation of maintained representations. This result also aligns with the broader view that CFC facilitates interactions between neural oscillations to coordinate complex cognitive operations, including those required for active restructuring of information (Abubaker et al., 2021; Hyafil et al., 2015; Pina et al., 2018). Furthermore, the differential CFC variability and network patterns also reflect that the differences in maintenance and manipulation are beyond quantitative strength.

Beyond its role in cognitive computation, the CFC findings in this study also offer insight into the functional characteristics of cognitive processing. The observed CFC effects in the central and parietal areas align with previous research identifying these areas as integral to high-level visual processing and spatial attention (Corbetta & Shulman, 2002; Koenigs et al., 2009). Related evidence suggests that dorsal-stream mechanisms are involved in the coordination and sequencing of visual information (Hebart & Hesselmann, 2012; Ries et al., 2019), processes that conceptually resemble the organizational demands engaged during information manipulation. The spatial pattern of CFC observed here may, therefore, reflect functional mechanisms tuned to the organizational demands of information manipulation, focusing more on relational and contextual processing than on the encoding of fine-grained detail. However, this interpretation remains coarse-grained and generic as scalp EEG data are limited in their ability to infer neural anatomical sources—an issue that awaits further investigation using fMRI or intracranial EEG.

Finally, the additional elicitation of functional neural networks during manipulation further demonstrates that WM manipulation involves distinct neural computational mechanisms. Functional neural networks indicate cross-regional coordination and control processes, which are essential for information manipulation. The functional networks observed in our work are characterized by directional features—specifically, the modulation of low-frequency activity at one electrode to high-frequency activity at another. The directionality and cross-electrode modulation indicate long-distance, cross-regional control and coordination, rather than simple synchronization or signal spread due to volume conduction. Based on these directional features, the distinct network patterns observed during manipulation provide strong neural evidence that the manipulation construct is functionally and mechanistically separable from maintenance.

### 4.5 Advantages, novelties, and limitations of the new WM tasks

#### 4.5.1 Advantages and novelties

The new task paradigms designed in the current study offer several advantages and novelties that contribute to a more nuanced investigation of WM processes. First, the tasks attempted to avoid potential strategy use and background knowledge effects. This was achieved by using uncommon visual patterns. Second, the task contents were designed to avoid the potential contribution of specific skills, such as verbal and arithmetic abilities or spatial cognition, to the behavioral performance. Third, and most importantly, the tasks were specifically designed to compare WM maintenance and manipulation within a unified paradigm.

#### 4.6.2 Limitations

Despite the advantages, certain limitations emerged during the implementation of the study. One notable limitation is its focus on the visual modality. Although the stimulus contents were specifically designed to be general and not dependent on knowledge, familiarity, and specific skills, the findings are limited to visual modalities and the current design. Participants may still employ a certain degree of complex visual and verbal encoding during the task performance. Further research, such as using auditory modality and employing more non-stimulus dependent content, is needed to determine whether the structure and psychometric characteristics of the WM constructs observed here are consistent across different sensory and cognitive domains.

A second limitation concerns task design. In the current version, three images are presented sequentially, and participants are asked to reorder them according to a specified sequence. This setup, with only three options available, might lead to participants’ use of inference strategies. Specifically, they may only memorize two images and infer the third. Although such strategy also involves information manipulation in principle, its use introduces a certain degree of heterogeneity into cross-individual performance variability. We received occasional reports of the participants using this strategy. To address this issue, future versions of the task could include four images—one of which is not part of the original sequence—as response options. This modification would rule out exclusion-based strategies and provide a more accurate reflection of individual differences in the cognitive abilities involved in WM maintenance and manipulation.

Additionally, in the operation span task, RT may be confounded by visual search demands, as participants must scan a matrix of letters before selecting a response. Since this factor was not controlled in the current study, some RT variability may reflect visual search rather than WM-specific processes.

There may be many other types of WM manipulation that exhibit different properties compared to the manipulation component examined in the current task. That said, this study is not sufficient to claim that WM consists of two unitary components as described. It merely aims to compare two components belonging to the broad categories of maintenance and manipulation and examine their distinct characteristics at both behavioral and neural levels. Other manipulation-like processes could exist, such as removing old items, updating newly encoded items, or inhibiting task-irrelevant items. Some of these processes may even overlap with other constructs, such as cognitive control. We acknowledge the complexity of the entire WM system and do not claim that this study definitively demonstrates a binary structure of WM. Instead, we focused on one representative contrast between maintenance and manipulation to explore their distinct characteristics at both behavioral and neural levels. In this context, the present study implemented manipulation using a sequence reordering task, which represents only one specific form of WM manipulation. This design choice may not fully capture the broader range of manipulation processes described in the literature (Ecker et al., 2010; Wager & Smith, 2003). Nonetheless, our aim was not to cover all possible forms of manipulation, but to use a clearly defined and tractable example to illustrate that maintenance and manipulation can be empirically distinguished and separately examined within a common framework. By focusing on a single, well-defined manipulation type, this study contributes to revealing the heterogeneity of the WM system, as supported by multi-level evidence. Moreover, demonstrating differences in the behavioral and neural characteristics of two tasks focused on maintenance and manipulation does not imply that the two WM components are fully separable in task implementation—it is not feasible in practice to design a task that purely involves maintenance or manipulation.

A further methodological limitation is that MI was computed from gamma-band activity measured with scalp EEG, where high-frequency signals are inherently noisier and more susceptible to non-neural contamination (e.g., muscle activity). Accordingly, gamma-related contributions to MI should be interpreted with caution when evaluating the CFC results.

Lastly, this study is based on EEG data from only 32 electrodes, which limits the precision of inferences about the neural sources of the observed effects. The data indicated that the relevant effects were highly localized over the central-parietal scalp region. However, this spatial resolution of the current EEG data is insufficient to provide strong evidence for their exact cortical origins.

## 5 Conclusion and Prospects

This study supports the view that WM can be separated into two distinct functional modules: maintenance and manipulation, which is in line with the contemporary conceptualization of WM. In addition, the dissociation between the newly designed tasks and traditional WM tasks reveals an important issue in WM research: its measurement is likely to be influenced by multiple extraneous factors. The fact that different tasks that are oriented to measuring the same construct (here, WM) can result in diverse cross-individual variability suggests that complex tasks may be susceptible to contamination by non-central processes, highlighting the challenges in designing tasks that effectively isolate WM-specific abilities. There remains a need for a more refined WM task system or battery that minimizes the influence of confounding variables and yields a purer measure of WM constructs.

The role of CFC as a neural indicator in WM is also noteworthy. Rather than being a general marker for information processing, CFC appears to be particularly associated with more complex WM-related processes. This differential association makes CFC a potential candidate for understanding the computational mechanisms underlying different levels of information processing in the brain.

Future research should focus on refining WM task paradigms to further reduce contamination from extraneous processes and to reveal a more precise structure of central information processing constructs. This includes developing tasks across sensory modalities and integrating neural measures for cross-validation. Additionally, identifying robust neural indicators, such as CFC, may offer a more direct approach for indexing and improving WM performance. Leveraging CFC as a tool for WM assessment could enable the creation of WM training paradigms that are immune to familiarity effects commonly affecting behavioral assessments in WM training studies. These efforts could inform the development of targeted WM training paradigms, addressing ongoing debates around the effectiveness of WM training and its transferability.

## Supplementary Material

Supplementary Material

## Data Availability

All anonymized behavioral data, EEG analysis scripts, and experimental protocols are publicly available on the Open Science Framework (OSF) at: https://osf.io/7bygx/overview.

## References

[IMAG.a.1155-b1] Aben, B., Stapert, S., & Blokland, A. (2012). About the distinction between working memory and short-term memory. Frontiers in Psychology, 3, 301. 10.3389/fpsyg.2012.00301 22936922 PMC3425965

[IMAG.a.1155-b2] Abubaker, M., Al Qasem, W., & Kvašňák, E. (2021). Working memory and cross-frequency coupling of neuronal oscillations. Frontiers in Psychology, 12, 756661. 10.3389/fpsyg.2021.756661 34744934 PMC8566716

[IMAG.a.1155-b3] Awh, E., Jonides, J., Smith, E. E., Schumacher, E. H., Koeppe, R. A., & Katz, S. (1996). Dissociation of storage and rehearsal in verbal working memory: Evidence from positron emission tomography. Psychological Science, 7(1), 25–31. 10.1111/j.1467-9280.1996.tb00662.x

[IMAG.a.1155-b4] Axmacher, N., Henseler, M. M., Jensen, O., Weinreich, I., Elger, C. E., & Fell, J. (2010). Cross-frequency coupling supports multi-item working memory in the human hippocampus. Proceedings of the National Academy of Sciences of the United States of America, 107(7), 3228–3233. 10.1073/pnas.0911531107 20133762 PMC2840289

[IMAG.a.1155-b5] Axmacher, N., Mormann, F., Fernández, G., Cohen, M. X., Elger, C. E., & Fell, J. (2007). Sustained neural activity patterns during working memory in the human medial temporal lobe. The Journal of Neuroscience: The Official Journal of the Society for Neuroscience, 27(29), 7807–7816. 10.1523/JNEUROSCI.0962-07.2007 17634374 PMC6672876

[IMAG.a.1155-b6] Baddeley, A. (1996). The fractionation of working memory. Proceedings of the National Academy of Sciences of the United States of America, 93(24), 13468–13472. 10.1073/pnas.93.24.13468 8942958 PMC33632

[IMAG.a.1155-b7] Baddeley, A. (2000). The episodic buffer: A new component of working memory? Trends in Cognitive Sciences, 4(11), 417–423. 10.1016/s1364-6613(00)01538-2 11058819

[IMAG.a.1155-b8] Baddeley, A. (2012). Working memory: Theories, models, and controversies. Annual Review of Psychology, 63, 1–29. 10.1146/annurev-psych-120710-100422 21961947

[IMAG.a.1155-b9] Bahramisharif, A., Jensen, O., Jacobs, J., & Lisman, J. (2018). Serial representation of items during working memory maintenance at letter-selective cortical sites. PLoS Biology, 16(8), e2003805. 10.1371/journal.pbio.2003805 30110320 PMC6093599

[IMAG.a.1155-b10] Berger, B., Griesmayr, B., Minarik, T., Biel, A. L., Pinal, D., Sterr, A., & Sauseng, P. (2019). Dynamic regulation of interregional cortical communication by slow brain oscillations during working memory. Nature Communications, 10(1), 4242. 10.1038/s41467-019-12057-0 PMC6751161 31534123

[IMAG.a.1155-b11] Brady, T. F., Störmer, V. S., & Alvarez, G. A. (2016). Working memory is not fixed-capacity: More active storage capacity for real-world objects than for simple stimuli. Proceedings of the National Academy of Sciences of the United States of America, 113(27), 7459–7464. 10.1073/pnas.1520027113 27325767 PMC4941470

[IMAG.a.1155-b12] Canolty, R. T., & Knight, R. T. (2010). The functional role of cross-frequency coupling. Trends in Cognitive Sciences, 14(11), 506–515. 10.1016/j.tics.2010.09.001 20932795 PMC3359652

[IMAG.a.1155-b13] Chun, M. M., Golomb, J. D., & Turk-Browne, N. B. (2011). A taxonomy of external and internal attention. Annual Review of Psychology, 62, 73–101. 10.1146/annurev.psych.093008.100427 19575619

[IMAG.a.1155-b14] Corbetta, M., & Shulman, G. L. (2002). Control of goal-directed and stimulus-driven attention in the brain. Nature Reviews. Neuroscience, 3(3), 201–215. 10.1038/nrn755 11994752

[IMAG.a.1155-b15] Cowan, N. (2001). The magical number 4 in short-term memory: A reconsideration of mental storage capacity. The Behavioral and Brain Sciences, 24(1), 87–185. 10.1017/s0140525x01003922 11515286

[IMAG.a.1155-b16] Cowan, N. (2010). The magical mystery four: How is working memory capacity limited, and why? Current Directions in Psychological Science, 19(1), 51–57. 10.1177/0963721409359277 20445769 PMC2864034

[IMAG.a.1155-b17] Cowan, N. (2014). Working memory underpins cognitive development, learning, and education. Educational Psychology Review, 26(2), 197–223. 10.1007/s10648-013-9246-y 25346585 PMC4207727

[IMAG.a.1155-b18] Cowan, N. (2017). The many faces of working memory and short-term storage. Psychonomic Bulletin & Review, 24(4), 1158–1170. 10.3758/s13423-016-1191-6 27896630

[IMAG.a.1155-b19] Crone, E. A., Wendelken, C., Donohue, S., van Leijenhorst, L., & Bunge, S. A. (2006). Neurocognitive development of the ability to manipulate information in working memory. Proceedings of the National Academy of Sciences of the United States of America, 103(24), 9315–9320. 10.1073/pnas.0510088103 16738055 PMC1472660

[IMAG.a.1155-b20] Daume, J., Kamiński, J., Schjetnan, A. G. P., Salimpour, Y., Khan, U., Kyzar, M., Reed, C. M., Anderson, W. S., Valiante, T. A., Mamelak, A. N., & Rutishauser, U. (2024). Control of working memory by phase-amplitude coupling of human hippocampal neurons. Nature, 629(8011), 393–401. 10.1038/s41586-024-07309-z 38632400 PMC11078732

[IMAG.a.1155-b21] Delorme, A., & Makeig, S. (2004). EEGLAB: An open source toolbox for analysis of single-trial EEG dynamics including independent component analysis. Journal of Neuroscience Methods, 134(1), 9–21. 10.1016/j.jneumeth.2003.10.009 15102499

[IMAG.a.1155-b22] D’Esposito, M., & Postle, B. R. (2015). The cognitive neuroscience of working memory. Annual Review of Psychology, 66, 115–142. 10.1146/annurev-psych-010814-015031 PMC4374359 25251486

[IMAG.a.1155-b23] D’Esposito, M., Postle, B. R., Ballard, D., & Lease, J. (1999). Maintenance versus manipulation of information held in working memory: An event-related fMRI study. Brain and Cognition, 41(1), 66–86. 10.1006/brcg.1999.1096 10536086

[IMAG.a.1155-b24] Draheim, C., Hicks, K. L., & Engle, R. W. (2016). Combining reaction time and accuracy: The relationship between working memory capacity and task switching as a case example. Perspectives on Psychological Science: A Journal of the Association for Psychological Science, 11(1), 133–155. 10.1177/1745691615596990 26817730

[IMAG.a.1155-b25] Ecker, U. K. H., Lewandowsky, S., Oberauer, K., & Chee, A. E. H. (2010). The components of working memory updating: An experimental decomposition and individual differences. Journal of Experimental Psychology: Learning, Memory, and Cognition, 36(1), 170–189. 10.1037/a0017891 20053053

[IMAG.a.1155-b26] Engle, R. W., Tuholski, S. W., Laughlin, J. E., & Conway, A. R. A. (1999). WM, short-term memory, and general fluid intelligence: A latent-variable approach. Journal of Experimental Psychology. General, 128(3), 309–331. 10.1037//0096-3445.128.3.309 10513398

[IMAG.a.1155-b27] Eriksson, J., Vogel, E. K., Lansner, A., Bergström, F., & Nyberg, L. (2015). Neurocognitive architecture of working memory. Neuron, 88(1), 33–46. 10.1016/j.neuron.2015.09.020 26447571 PMC4605545

[IMAG.a.1155-b28] Friedman, N. P., & Miyake, A. (2017). Unity and diversity of executive functions: Individual differences as a window on cognitive structure. Cortex; a Journal Devoted to the Study of the Nervous System and Behavior, 86, 186–204. 10.1016/j.cortex.2016.04.023 27251123 PMC5104682

[IMAG.a.1155-b29] Gazzaley, A., & Nobre, A. C. (2012). Top-down modulation: Bridging selective attention and working memory. Trends in Cognitive Sciences, 16(2), 129–135. 10.1016/j.tics.2011.11.014 22209601 PMC3510782

[IMAG.a.1155-b30] Glahn, D. C., Kim, J., Cohen, M. S., Poutanen, V. P., Therman, S., Bava, S., Van Erp, T. G., Manninen, M., Huttunen, M., Lönnqvist, J., Standertskjöld-Nordenstam, C. G., & Cannon, T. D. (2002). Maintenance and manipulation in spatial working memory: Dissociations in the prefrontal cortex. NeuroImage, 17(1), 201–213. 10.1006/nimg.2002.1161 12482077

[IMAG.a.1155-b31] Hebart, M. N., & Hesselmann, G. (2012). What visual information is processed in the human dorsal stream? The Journal of Neuroscience: The Official Journal of the Society for Neuroscience, 32(24), 8107–8109. 10.1523/JNEUROSCI.1462-12.2012 22699890 PMC6703654

[IMAG.a.1155-b32] Heitz R. P. (2014). The speed-accuracy tradeoff: History, physiology, methodology, and behavior. Frontiers in Neuroscience, 8, 150. 10.3389/fnins.2014.00150 24966810 PMC4052662

[IMAG.a.1155-b33] Hinson, J. M., Jameson, T. L., & Whitney, P. (2003). Impulsive decision making and working memory. Journal of Experimental Psychology: Learning, Memory, and Cognition, 29(2), 298–306. 10.1037/0278-7393.29.2.298 12696817

[IMAG.a.1155-b34] Holz, E. M., Glennon, M., Prendergast, K., & Sauseng, P. (2010). Theta-gamma phase synchronization during memory matching in visual working memory. NeuroImage, 52(1), 326–335. 10.1016/j.neuroimage.2010.04.003 20382239

[IMAG.a.1155-b35] Hwang, G., Jacobs, J., Geller, A., Danker, J., Sekuler, R., & Kahana, M. J. (2005). EEG correlates of verbal and nonverbal working memory. Behavioral and Brain Functions: BBF, 1, 20. 10.1186/1744-9081-1-20 16287504 PMC1315326

[IMAG.a.1155-b36] Hyafil, A., Giraud, A. L., Fontolan, L., & Gutkin, B. (2015). Neural cross-frequency coupling: Connecting architectures, mechanisms, and functions. Trends in Neurosciences, 38(11), 725–740. 10.1016/j.tins.2015.09.001 26549886

[IMAG.a.1155-b37] Jablonska, K., Piotrowska, M., Bednarek, H., Szymaszek, A., Marchewka, A., Wypych, M., & Szelag, E. (2020). Maintenance vs. manipulation in auditory verbal working memory in the elderly: New insights based on temporal dynamics of information processing in the millisecond time range. Frontiers in Aging Neuroscience, 12, 194. 10.3389/fnagi.2020.00194 32848698 PMC7396649

[IMAG.a.1155-b38] Jasper, H. H. (1958) The ten-twenty electrode system of the international federation. Electroencephalography and Clinical Neurophysiology, 10, 371–375. 10.1016/0013-4694(58)90051-8 10590970

[IMAG.a.1155-b39] Jirsa, V., & Müller, V. (2013). Cross-frequency coupling in real and virtual brain networks. Frontiers in Computational Neuroscience, 7, 78. 10.3389/fncom.2013.00078 23840188 PMC3699761

[IMAG.a.1155-b40] Jones, G., & Macken, B. (2015). Questioning short-term memory and its measurement: Why digit span measures long-term associative learning. Cognition, 144, 1–13. 10.1016/j.cognition.2015.07.009 26209910

[IMAG.a.1155-b41] Jones, K. T., Johnson, E. L., & Berryhill, M. E. (2020). Frontoparietal theta-gamma interactions track working memory enhancement with training and tDCS. NeuroImage, 211, 116615. 10.1016/j.neuroimage.2020.116615 32044440 PMC7733399

[IMAG.a.1155-b42] Kane, M. J., Hambrick, D. Z., Tuholski, S. W., Wilhelm, O., Payne, T. W., & Engle, R. W. (2004). The generality of working memory capacity: A latent-variable approach to verbal and visuospatial memory span and reasoning. Journal of Experimental Psychology. General, 133(2), 189–217. 10.1037/0096-3445.133.2.189 15149250

[IMAG.a.1155-b43] Kirchner, W. K. (1958). Age differences in short-term retention of rapidly changing information. Journal of Experimental Psychology, 55(4), 352–358. 10.1037/h0043688 13539317

[IMAG.a.1155-b44] Kiyonaga, A., & Egner, T. (2013). Working memory as internal attention: Toward an integrative account of internal and external selection processes. Psychonomic Bulletin & Review, 20(2), 228–242. 10.3758/s13423-012-0359-y 23233157 PMC3594067

[IMAG.a.1155-b45] Koenigs, M., Barbey, A. K., Postle, B. R., & Grafman, J. (2009). Superior parietal cortex is critical for the manipulation of information in working memory. The Journal of Neuroscience: The Official Journal of the Society for Neuroscience, 29(47), 14980–14986. 10.1523/JNEUROSCI.3706-09.2009 19940193 PMC2799248

[IMAG.a.1155-b46] Lisman, J. E., & Idiart, M. A. (1995). Storage of 7 +/- 2 short-term memories in oscillatory subcycles. Science (New York, N.Y.), 267(5203), 1512–1515. 10.1126/science.7878473 7878473

[IMAG.a.1155-b47] Lisman, J. E., & Jensen, O. (2013). The θ-γ neural code. Neuron, 77(6), 1002–1016. 10.1016/j.neuron.2013.03.007 23522038 PMC3648857

[IMAG.a.1155-b48] Masse, N. Y., Yang, G. R., Song, H. F., Wang, X. J., & Freedman, D. J. (2019). Circuit mechanisms for the maintenance and manipulation of information in working memory. Nature Neuroscience, 22(7), 1159–1167. 10.1038/s41593-019-0414-3 31182866 PMC7321806

[IMAG.a.1155-b49] Meule A. (2017). Reporting and interpreting working memory performance in n-back tasks. Frontiers in Psychology, 8, 352. 10.3389/fpsyg.2017.00352 28326058 PMC5339218

[IMAG.a.1155-b50] Miller, E. K., & Cohen, J. D. (2001). An integrative theory of prefrontal cortex function. Annual Review of Neuroscience, 24, 167–202. 10.1146/annurev.neuro.24.1.167 11283309

[IMAG.a.1155-b51] Miller, E. K., Lundqvist, M., & Bastos, A. M. (2018). Working Memory 2.0. Neuron, 100(2), 463–475. 10.1016/j.neuron.2018.09.023 30359609 PMC8112390

[IMAG.a.1155-b52] Mohr, H. M., Goebel, R., & Linden, D. E. (2006). Content- and task-specific dissociations of frontal activity during maintenance and manipulation in visual working memory. The Journal of Neuroscience: The Official Journal of the Society for Neuroscience, 26(17), 4465–4471. 10.1523/JNEUROSCI.5232-05.2006 16641225 PMC6674080

[IMAG.a.1155-b53] Oberauer, K. (2009). Design for a working memory. In B. H. Ross (Ed.), The psychology of learning and motivation (pp. 45–100). Elsevier Academic Press. 10.1016/S0079-7421(09)51002-X

[IMAG.a.1155-b54] Oberauer, K. (2019). Working memory and attention—A conceptual analysis and review. Journal of Cognition, 2(1), Article 36. 10.5334/joc.58 PMC6688548 31517246

[IMAG.a.1155-b55] Ouyang, G., & Li, Y. (2025). Protocol for semi-automatic EEG preprocessing incorporating independent component analysis and principal component analysis. STAR Protocols, 6(1), 103682. 10.1016/j.xpro.2025.103682 40053447 PMC11930125

[IMAG.a.1155-b56] Owen, A. M., McMillan, K. M., Laird, A. R., & Bullmore, E. (2005). N-back working memory paradigm: A meta-analysis of normative functional neuroimaging studies. Human Brain Mapping, 25(1), 46–59. 10.1002/hbm.20131 15846822 PMC6871745

[IMAG.a.1155-b57] Phillips, W. A., & Baddeley, A. D. (1971). Reaction time and short-term visual memory. Psychonomic Science, 22(2), 73–74. 10.3758/BF03332500

[IMAG.a.1155-b58] Pina, J. E., Bodner, M., & Ermentrout, B. (2018). Oscillations in working memory and neural binding: A mechanism for multiple memories and their interactions. PLoS Computational Biology, 14(11), e1006517. 10.1371/journal.pcbi.1006517 30419015 PMC6258380

[IMAG.a.1155-b59] Pion-Tonachini, L., Kreutz-Delgado, K., & Makeig, S. (2019). ICLabel: An automated electroencephalographic independent component classifier, dataset, and website. NeuroImage, 198, 181–197. 10.1016/j.neuroimage.2019.05.026 31103785 PMC6592775

[IMAG.a.1155-b60] Riddle, J., McFerren, A., & Frohlich, F. (2021). Causal role of cross-frequency coupling in distinct components of cognitive control. Progress in Neurobiology, 202, 102033. 10.1016/j.pneurobio.2021.102033 33741402 PMC8184612

[IMAG.a.1155-b61] Ries, S. K., Piai, V., Perry, D., Griffin, S., Jordan, K., Henry, R., Knight, R. T., & Berger, M. S. (2019). Roles of ventral versus dorsal pathways in language production: An awake language mapping study. Brain and Language, 191, 17–27. 10.1016/j.bandl.2019.01.001 30769167 PMC6402581

[IMAG.a.1155-b62] Roberts, B. M., Hsieh, L. T., & Ranganath, C. (2013). Oscillatory activity during maintenance of spatial and temporal information in working memory. Neuropsychologia, 51(2), 349–357. 10.1016/j.neuropsychologia.2012.10.009 23084981 PMC3546228

[IMAG.a.1155-b63] Roux, F., & Uhlhaas, P. J. (2014). Working memory and neural oscillations: Alpha-gamma versus theta-gamma codes for distinct WM information? Trends in Cognitive Sciences, 18, 16–25. 10.1016/j.tics.2013.10.010 24268290

[IMAG.a.1155-b64] Sauseng, P., Peylo, C., Biel, A. L., Friedrich, E. V. C., & Romberg-Taylor, C. (2019). Does cross-frequency phase coupling of oscillatory brain activity contribute to a better understanding of visual working memory? British Journal of Psychology (London, England: 1953), 110(2), 245–255. 10.1111/bjop.12340 30079531

[IMAG.a.1155-b65] Seo, J., Lee, B. K., Jin, S. U., Park, J. W., Kim, Y. T., Ryeom, H. K., Lee, J., Suh, K. J., Kim, S. H., Park, S. J., Jeong, K. S., Ham, J. O., Kim, Y., & Chang, Y. (2014). Lead-induced impairments in the neural processes related to working memory function. PLoS One, 9(8), e105308. 10.1371/journal.pone.0105308 25141213 PMC4139362

[IMAG.a.1155-b66] Shi, D., & Yu, Q. (2024). Distinct neural signatures underlying information maintenance and manipulation in working memory. Cerebral Cortex (New York, N.Y.: 1991), 34(3), bhae063. 10.1093/cercor/bhae063 38436467

[IMAG.a.1155-b67] Smith, E. E., & Jonides, J. (1999). Storage and executive processes in the frontal lobes. Science (New York, N.Y.), 283(5408), 1657–1661. 10.1126/science.283.5408.1657 10073923

[IMAG.a.1155-b68] Stuart, G., & Hulme, C. (2000). The effects of word co-occurrence on short-term memory: Associative links in long-term memory affect short-term memory performance. Journal of Experimental Psychology. Learning, Memory, and Cognition, 26(3), 796–802. 10.1037//0278-7393.26.3.796 10855432

[IMAG.a.1155-b69] Süß, H.-M., Oberauer, K., Wittmann, W. W., Wilhelm, O., & Schulze, R. (2002). Working-memory capacity explains reasoning ability—And a little bit more. Intelligence, 30(3), 261–288. 10.1016/S0160-2896(01)00100-3

[IMAG.a.1155-b70] Swanson, L., & Kim, K. (2007). WM, short-term memory, and naming speed as predictors of children’s mathematical performance. Intelligence, 35(2), 151–168. 10.1016/j.intell.2006.07.001

[IMAG.a.1155-b71] Tamura, M., Spellman, T. J., Rosen, A. M., Gogos, J. A., & Gordon, J. A. (2017). Hippocampal-prefrontal theta-gamma coupling during performance of a spatial working memory task. Nature Communications, 8(1), 2182. 10.1038/s41467-017-02108-9 PMC5736608 29259151

[IMAG.a.1155-b72] Tort, A. B., Komorowski, R., Eichenbaum, H., & Kopell, N. (2010). Measuring phase-amplitude coupling between neuronal oscillations of different frequencies. Journal of Neurophysiology, 104(2), 1195–1210. 10.1152/jn.00106.2010 20463205 PMC2941206

[IMAG.a.1155-b73] Tort, A. B., Komorowski, R. W., Manns, J. R., Kopell, N. J., & Eichenbaum, H. (2009). Theta-gamma coupling increases during the learning of item-context associations. Proceedings of the National Academy of Sciences of the United States of America, 106(49), 20942–20947. 10.1073/pnas.0911331106 19934062 PMC2791641

[IMAG.a.1155-b74] Turner, M. L., & Engle, R. W. (1989). Is working memory capacity task dependent? Journal of Memory and Language, 28(2), 127–154. 10.1016/0749-596X(89)90040-5

[IMAG.a.1155-b75] Unsworth, N., & Engle, R. W. (2005). Working memory capacity and fluid abilities: Examining the correlation between Operation Span and Raven. Intelligence, 33(1), 67–81. 10.1016/j.intell.2004.08.003

[IMAG.a.1155-b76] Vandierendonck, A. (2021). Multicomponent working memory system with distributed executive control. In R. H. Logie, V. Camos, & N. Cowan (Eds.), Working memory: State of the science (pp. 150–174). Oxford University Press. 10.1093/oso/9780198842286.003.0006

[IMAG.a.1155-b77] Van Hecke, J., Gladwin, T. E., Coremans, J., Destoop, M., Hulstijn, W., & Sabbe, B. (2010). Prefrontal, parietal and basal activation associated with the reordering of a two-element list held in working memory. Biological Psychology, 85(1), 143–148. 10.1016/j.biopsycho.2010.06.005 20542080

[IMAG.a.1155-b78] Veltman, D. J., Rombouts, S. A., & Dolan, R. J. (2003). Maintenance versus manipulation in verbal working memory revisited: An fMRI study. NeuroImage, 18(2), 247–256. 10.1016/s1053-8119(02)00049-6 12595179

[IMAG.a.1155-b79] Vergauwe, E., Camos, V., & Barrouillet, P. (2014). The impact of storage on processing: How is information maintained in working memory? Journal of Experimental Psychology: Learning, Memory, and Cognition, 40(4), 1072–1095. 10.1037/a0035779 24564542

[IMAG.a.1155-b80] Wager, T. D., & Smith, E. E. (2003). Neuroimaging studies of working memory: A meta-analysis. Cognitive, Affective & Behavioral Neuroscience, 3(4), 255–274. 10.3758/cabn.3.4.255 15040547

[IMAG.a.1155-b82] Wiley, J., & Jarosz, A. F. (2012). How working memory capacity affects problem solving. In B. H. Ross (Ed.), The psychology of learning and motivation (pp. 185–227). Elsevier Academic Press. 10.1016/B978-0-12-394393-4.00006-6

[IMAG.a.1155-b83] Wilhelm, O., Hildebrandt, A., & Oberauer, K. (2013). What is working memory capacity, and how can we measure it? Frontiers in Psychology, 4, 433. 10.3389/fpsyg.2013.00433 23898309 PMC3721021

