# Supplementary material for: Behavioral variability and neural cross-frequency coupling distinguish working memory maintenance from manipulation
Source: Imaging Neurosci (Camb). 2026 Feb 27;4:IMAG.a.1155. doi: 10.1162/IMAG.a.1155 (PMC12951641; doi:10.1162/IMAG.a.1155)
Supplement: Supplementary Material [file IMAG.a.1155_supp.pdf]

Supplementary Materials

Table S1. Split-Half Confirmatory Factor Analysis Results

Two Factor Model
Standardized factor loadings. Significance: * *$p < 0.05$; ** $p < 0.01$; *** $p < 0.001$*

Indicator	Split 1 Loading	Split 2 Loading
MT Indicator 1	0.55***	0.70***
MT Indicator 2	0.58***	0.70***
MT Indicator 3	0.77***	0.66***
MT Indicator 4	0.58***	0.73***
MP Indicator 1	0.81***	0.58***
MP Indicator 2	0.78***	0.62***
MP Indicator 3	0.61***	0.66***
MP Indicator 4	0.62***	0.86***

Inter-factor covariance (r):
Split one: 0.62
Split two: 0.43

One Factor Model
Standardized factor loadings. Significance: * *$p < 0.05$; ** $p < 0.01$; *** $p < 0.001$*

Indicator	Split 1 Loading	Split 2 Loading
Indicator 1	0.44**	0.61***
Indicator 2	0.54***	0.62***
Indicator 3	0.54***	0.70***
Indicator 4	0.40**	0.55***
Indicator 5	0.80***	0.40*
Indicator 6	0.74***	0.56***
Indicator 7	0.61***	0.66***
Indicator 8	0.61***	0.55***

Model Comparison (two-factor vs one-factor): split 1: $\Delta\chi^2(1) = 18.96$, $p < 0.001$; split 2: $\Delta\chi^2(1) = 35.84$, $p < 0.001$.

Table S2. Split-Half Exploratory Factor Analysis Results

Split 1
Standardized factor loadings. Significance: * *$p < 0.05$; ** $p < 0.01$; *** $p < 0.001$*

Indicator	Factor 1	Factor 2
MT Indicator 1	0.39*	0.17
MT Indicator 2	0.37*	0.30

MT Indicator 3	0.88***	-0.01
MT Indicator 4	0.59***	0.01
MP Indicator 1	0.09	0.77***
MP Indicator 2	-0.02	0.80***
MP Indicator 3	0.15	0.53***
MP Indicator 4	-0.08	0.69***

Split 2

Standardized factor loadings. Significance: * *p < 0.05; *** *p < 0.01;* *** *p < 0.001*

Indicator	Factor 1	Factor 2
MT Indicator 1	0.68***	0.03
MT Indicator 2	0.72***	-0.03
MT Indicator 3	0.70***	0.03
MT Indicator 4	0.70***	-0.04
MP Indicator 1	0.05	0.55***
MP Indicator 2	0.26*	0.47***
MP Indicator 3	0.33**	0.48***
MP Indicator 4	-0.02	0.99***

Table S3. Path Analysis Results among Behavioral Variables

Standardized factor loadings. Significance: * *p < 0.05; *** *p < 0.01;* *** *p < 0.001*

Path	Estimate	SE	z-value	Std. β
SM -> MT	0.41***	0.11	3.72	0.41***
SM -> MP	0.12	0.12	0.98	0.12
MT <-> MP	0.61***	0.12	0.51	0.61***
MT <-> OS	0.27*	0.11	2.36	0.27*
MP <-> OS	0.28*	0.12	2.28	0.28*
SM <-> OS	0.06	0.13	4.63	0.06

Table S4. Split-Half Structural Equation Modeling Results for Behavioral Relationships

Standardized factor loadings. Significance: * *p < 0.05; *** *p < 0.01;* *** *p < 0.001*

Path	Estimate (Split 1)	Std. β (Split 1)	Estimate (Split 2)	Std. β (Split 2)
SM -> MT	0.30	0.29	0.44**	0.40**
SM -> MP	-0.04	-0.04	0.04	0.04
MT <-> MP	0.65***	0.65***	0.45**	0.45**
MT <-> OS	0.02	0.02	0.25	0.25

MP <-> OS	0.15	0.15	0.26	0.26
SM <-> OS	-0.07	-0.07	-0.06	-0.06

Table S5. Split-Half Structural Equation Modeling Results for MI-RT Models

Standardized factor loadings. Significance: * *p < 0.05; ** p < 0.01; *** p < 0.001*

(A) Maintenance Model

Parameter	Split 1	Split 2
**Path Coefficient**		
Neural CFC -> Behavioral RT	-0.17	0.18
**MI Factor Loadings**		
Indicator 1	0.27	0.46*
Indicator 2	0.32	0.55**
Indicator 3	1.30	0.47*
**RT Factor Loadings**		
Indicator 1	0.52***	0.71***
Indicator 2	0.53***	0.69***
Indicator 3	0.80***	0.64***
Indicator 4	0.62***	0.77***

(B) Manipulation Model

Parameter	Split 1	Split 2
**Path Coefficient**		
Neural CFC -> Behavioral RT	-0.38*	-0.38*
**MI Factor Loadings**		
Indicator 1	0.62***	0.67***
Indicator 2	0.66***	0.61***
Indicator 3	0.78***	0.79***
**RT Factor Loadings**		
Indicator 1	0.80***	0.58***
Indicator 2	0.80***	0.61***
Indicator 3	0.59***	0.63***
Indicator 4	0.62***	0.88***

Figure S1. Confirmatory factor models for intra-task and inter-task analyses of individual variability

Schematic illustration of the one-factor and two-factor models used to test the latent structure of MI variability. For each participant, MI values were divided into lower- and higher-magnitude subsets, with the order within each subset randomly permuted before averaging into three indicators per subset.

Panel A-D show the WM maintenance (A-B) and manipulation (C-D) conditions, each with one-factor (A, C) and two-factor (B, D) models. These models test intra-task stability across low- and high-MI subsets.

Panels E-F compare the maintenance and manipulation conditions jointly (inter-task analysis), with Panel E showing the two-factor model and Panel F the one-factor model.

Corresponding fit indices and χ^2 - difference tests are provided in Table S6.

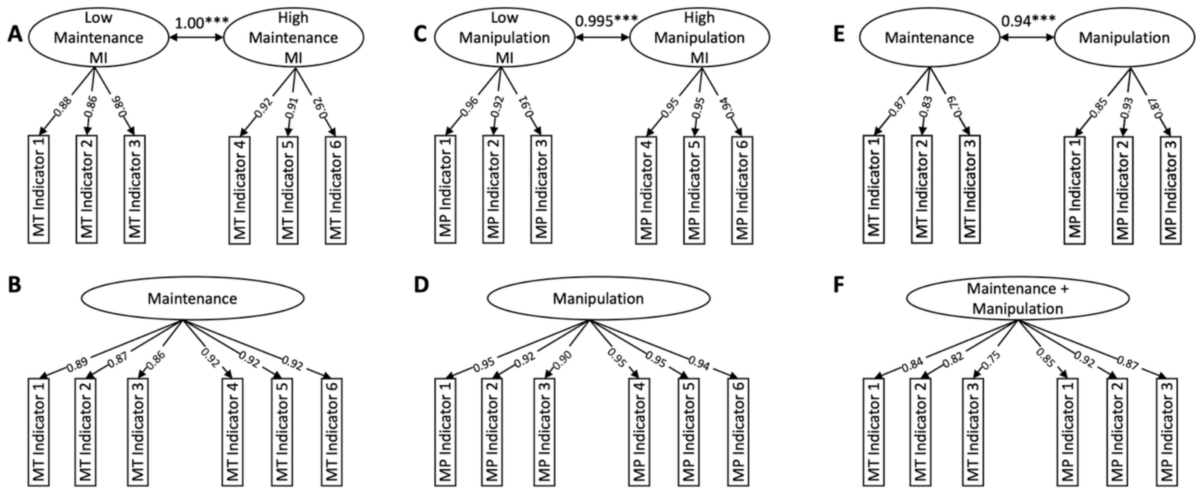

Table S6. Model fit indices and χ^2-difference tests for intra-task and inter-task analyses of individual variability

condition	Model	χ^2 (df)	RMSEA [90 % CI]	CFI	$\Delta\chi^2$ (Δ df)	*p*
WM Maintenance	One-factor	17.79 (9)	0.12 [0.03-0.21]	0.98	–	–
	Two-factor	16.85 (8)	0.13 [0.04-0.22]	0.98	0.94 (1)	0.33
WM Manipulation	One-factor	13.70 (9)	0.09 [0.00-0.18]	0.99	–	–
	Two-factor	13.30 (8)	0.10 [0.00-0.20]	0.99	0.41 (1)	0.52
Maintenance vs Manipulation	One-factor	15.03 (9)	0.10 [0.00-0.19]	0.98	–	–
	Two-factor	9.49 (8)	0.05 [0.00-0.16]	1.00	5.54 (1)	**0.019***
